# Selective neuronal restoration of progranulin does not prevent the frontotemporal dementia like-phenotype of progranulin knockout mice

**DOI:** 10.1186/s12974-025-03665-3

**Published:** 2026-01-10

**Authors:** Marc-Philipp Weyer, Lisa Hahnefeld, Luisa Franck, Carlo Angioni, Matthias Klein, Gerd Geisslinger, Michael K.E. Schäfer, Irmgard Tegeder

**Affiliations:** 1 Institute of Clinical Pharmacology, Faculty of Medicine, Goethe University Frankfurt, Theodor-Stern-Kai 7, 60590 Frankfurt am Main, Germany; 2 Fraunhofer Institute for Translational Medicine and Pharmacology ITMP and Fraunhofer Cluster of Excellence for Immune Mediated Diseases CIMD, Theodor-Stern-Kai 7, Frankfurt am Main, 60596 Germany; 3 Institute of Immunology, University Medical Center, Johannes Gutenberg-University Mainz, Langenbeckstr. 1, 55131 Mainz, Germany; 4 Department of Anaesthesiology, University Medical Center, Johannes Gutenberg- University, 55131 Mainz, Germany; 5 Focus Program Translational Neurosciences (FTN) of the Johannes Gutenberg-University, Mainz, 55121 Germany

**Keywords:** Frontotemporal dementia, Progranulin, Microglia, Lipidomic and metabolomic analyses, Lysosomes, IntelliCage, Behaviour

## Abstract

Progranulin (PGRN) is a neurotrophic and anti-inflammatory factor produced mainly by neurons and microglia in the central nervous system. Progranulin haploinsufficiency causes frontotemporal dementia (FTD). It is unclear to what extent neuronal versus microglial PGRN deficiency contributes to FTD pathology. In this study, we restored progranulin in neurons in progranulin knockout mice using Nestin-driven expression of mouse *Grn* transgene in a *k*nock*o*ut *b*ack*g*round (NesGrn KOBG). They were compared with full PGRN KO mice and floxed control mice that carry a loxP flanked STOP codon in front of m*Grn* transgene (Grn-flfl). The expected neuron-only PGRN rescue was confirmed at RNA and protein level in brain tissue and primary cells, and single nucleus RNA sequencing. Despite neuronal PGRN-restoration, there was no difference in microgliosis, astrogliosis, and microglia phenotypes as assessed by histology, microglia morphometry and bulk RNAseq showing strong upregulation of microglia-associated genes equally in both KO lines. However, a microglial subpopulation with a phagocyte signature expressing *Gpnmb*,* Lgals3*,* Atp6v0d2* and *Apobec1* occurred only in PGRN KO brain, and accordingly, the loss of synapses and dendritic spines, which is caused by excessive synaptic pruning in PGRN KO mice, was partially attenuated in NesGrn KOBG mice. Lipidomic studies showed that phosphatidylserine eat-me-signals were increased in PGRN KO but not in NesGrn KOBG brain. Furthermore, some neuronal genes involved in axonal structure and dynamics were co-restored with progranulin in NesGrn KOBG mice. However, the modest improvement of neuronal health was not associated with an improvement of FTD-like behavior including hyperactivity, compulsive licking and impaired avoidance learning and memory. The results suggest that (still) viable neurons do not provide (sufficient) progranulin to prevent microgliosis but may shape the phenotype by presenting or hiding eat-me signals. Nonetheless, neuron-only-progranulin restoration may be insufficient to halt the progression of FTD.

**Supplementary Information:**

The online version contains supplementary material available at 10.1186/s12974-025-03665-3.

## Introduction

Progranulin (PGRN) is a neurotrophic and anti-inflammatory protein expressed mainly by neurons and microglia in the CNS [1]. Its functions are mediated by extracellular signaling via cell surface receptors including Notch and EphA2 [2, 3], inhibition of TNFα receptor signaling [4] and internalization and endolysosomal sorting via Sortilin (SorCS2) [5]. Progranulin also promotes intracellular processes related to phagocytosis, autophagy, and lysosomal degradation [6, 7], in particular the autophagolysosomal flux. However, it is still unresolved how vesicular progranulin may operate from the inside of the lysosome to enhance its functionality. Early work suggested that progranulin needs to be secreted and subsequently re-internalized via the VPS10 domain-containing receptor sortilin [5] to reach the lysosome, while other more recent work has shown that lysosomal trafficking of PGRN occurs via intracellular routes [8].

Human loss-of-function mutations in the *GRN* gene are associated with neurodegenerative diseases, including frontotemporal dementia (FTD) and neuronal ceroid lipofuscinosis [9–12]. Studies in PGRN deficient mice support the therapeutic relevance of genetic or pharmacological approaches to restore or enhance the expression of PGRN [13–18]. The encouraging therapeutic effects of progranulin replacement either by adeno-associated virus-mediated brain [13, 17, 19] or peripheral delivery [20] or progranulin-like peptides [21] in preclinical models prompted clinical trials of progranulin gene therapy (NCT04747431, NCT04408625, and NCT06064890) or monoclonal sortilin antibody therapy (NCT04374136), the latter to increase extracellular PGRN levels by inhibiting its uptake [14, 22] under the assumption that progranulin’s effects are mediated through binding to the extracellular site of progranulin-responsive membrane bound receptors. However, the PGRN elevating monoclonal anti-sortilin antibody latozinemab failed to slow or reverse neurodegeneration-biomarker levels or the progressive cognitive decline despite increasing extracellular progranulin levels 2–3-fold (NCT03987295) [23]. The failure of anti-sortilin mAb emphasizes the importance of intracellular progranulin or sortilin-independent mechanisms of progranulin internalization, the latter supported by the success of a brain-penetrant progranulin-like derivative [21], which does not require sortilin.

Adeno associated virus-9 (AAV9) mediated *Grn* delivery also restores progranulin independent of sortilin, but only/mainly in neurons because AAV9 primarily targets neurons [24]. AAV9-*Grn* injected into the lateral ventricle resulted in a widespread expression of *Grn* in *Grn* null mouse brain. However, one study reported that the overexpression resulted in dramatic and selective hippocampal toxicity and degeneration affecting both neurons and glia [25]. In another study, intraventricular injection of PGRN-expressing AAV1/9 viruses partially rescued motor deficits, neuronal loss, glial activation, and lysosomal abnormalities in *Tmem106b-Grn* double knockout mice [13]. AAV9 but not AAV1-mediated expression of PGRN resulted in high levels of PGRN in the serum [13]. Further studies showed that AAV9-*Grn* mediated gene therapy in PGRN heterozygous (het) mice attenuated social inferiority behaviour in a tube test [16]. Although PGRN het mice theoretically mimic human haploinsufficiency of PGRN in FTD, they do not replicate the dominant negative effects of mutant *Grn*. They maintain about 50% of normal PGRN levels and are healthy in most aspects at the histology, molecular biology and at the behaviour level. Hence, normalization of social fear in a tube test does not necessarily tell if the approach was successful in age-dependent progression of FTD caused by *Grn* haploinsufficiency. Nonetheless, interim results of a first-in-human study with AAV9-*Grn* (PR006) showed that one-time administration of PR006 into the cisterna magna was generally safe and CSF progranulin increased in all patients. Anti-AAV9 antibodies emerged during treatment in all patients accompanied with pleocytosis in the CSF but so far, no anti-progranulin antibodies were found [24]. The data suggest that restoration of *Grn* in neurons is crucial for therapeutic efficacy, which is further supported indirectly by a study in which additional deletion of progranulin in myeloid cells (via Lyz-Cre) in a neuronal progranulin mouse model did not aggravate the phenotype of the Camk2a-Grn^−/−^ mice [26]. Unfortunately, it was not assessed if Lyz-Grn^−/−^ mono-microglia knockout mice develop a “dementia-like” phenotype.

In theory, the depletion of microglia followed by the transplantation of gene-edited microglia or stem cells, whether allogenic or autologous, may be feasible. This, however, would require an efficient repopulation and microglia-mediated supply of progranulin to neurons. It is not clear if neurons are able to replenish their own progranulin with the extracellular progranulin provided by microglia. In a pre-clinical mouse study, it was shown that an allogeneic bone marrow transplant to progranulin-deficient mice, which were conditioned with busulfan and PLX3397, restored progranulin in the brain and eyes and normalized brain lipofuscin, lysosomal functions, and lipid metabolism [27]. Case reports have shown that hematopoietic stem cell transplantation after busulfan conditioning can lead to a partial repopulation of microglia-like cells and can deliver therapeutic proteins to the CNS in human microglia-dependent genetic diseases [28–31]. However, current conditioning approaches result in low and slow engraftment of transplanted cells in the CNS. The transplantation studies suggested that microglial progranulin is as important as the neuronal progranulin, which may broaden the therapeutic options. Encouragingly, even a peripheral liver-AAV-mediated boost of a progranulin was found to cure progranulin-knockout mice [20]. In this study, fusion of progranulin with a transferrin receptor binding protein mediated the transfer through the blood brain barrier and cellular uptake of progranulin, both into neurons and microglia [20]. Because it is important for future directions of therapeutic progranulin-replacing strategies how and where progranulin is replaced, we created a mouse line with neuronal progranulin restoration on a full knockout background and studied behavior, neuroinflammation, molecular and metabolic features of these mice. Briefly, the results show a subtle improvement of neuronal health but no improvement of neuroinflammation and FTD-like behavior.

## Methods

### Animals

**Fig. 1:**
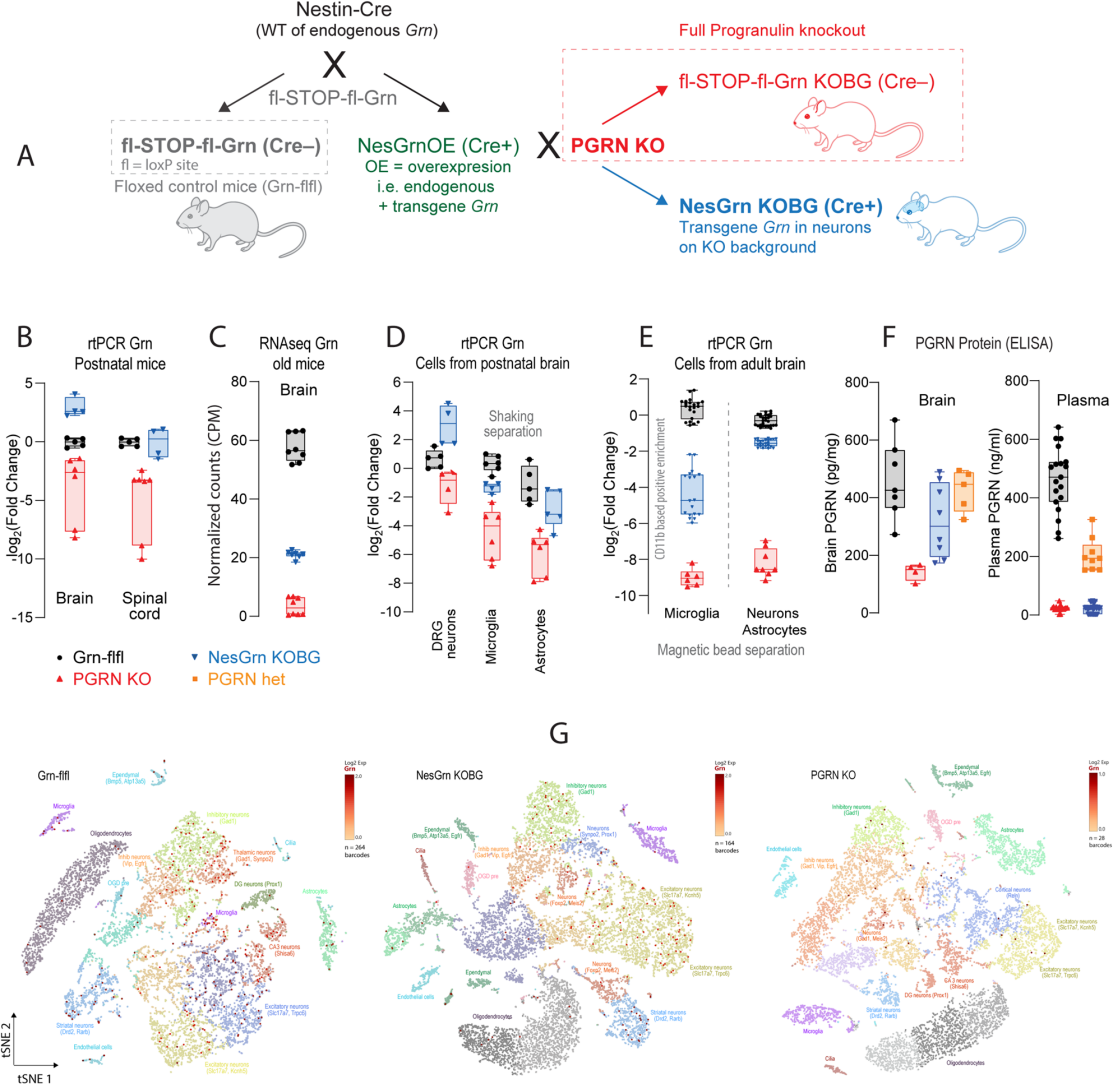
Expression of progranulin in NesGrn KOGB mice **A**: Graphical visualization of the mouse lines and breeding scheme. The loxP-STOP-loxP-Grn mice who carry the transgene inserted into the Rosa26 locus (referred to as Grn-flfl) were crossed with Nestin-Cre to delete the STOP codon in front of the mouse *Grn* transgene to generate NesGrnOE mice which express endogenous *Grn* plus *Grn* transgene (OE= overexpression). These mice were crossed with full progranulin knockout mice (PGRN KO) in which the endogenous *Grn* is deleted to create mice with neuronal expression of *Grn* in progranulin knockout background (NesGrn KOBG). Mice were bred to homozygosity of the knockout allele, the floxed transgene and hemizygosity for Cre. **B-E**: rtPCR or RNAseq analysis of *Grn* mRNA expression in neuronal tissue of postnatal (P1-3) and old mice (13-15 months), respectively and in isolated neurons, microglia and astrocytes of postnatal (P1-3) and old mice (avg. 8-10 months). Data are presented as box/scatter plots. The line is the median, the box shows the interquartile range, the whiskers show minimum to maximum, scatters show results of individual mice or cultures, n = 4-8 mice in **B, C, D**. In **E**, results are from 3-11 mice per group, measured in duplicates. **F**: PGRN protein ELISA analysis of brain homogenate and plasma samples of old mice (11-13 months). Data are presented as box/scatter plots. The line is the median, the box shows the interquartile range, the whiskers show minimum to maximum, scatters show results of individual mice, n = 5-8 mice per group for brain, n = 9-20 for plasma.**G**: t-SNE plots of single nucleus mRNA analysis of 12-24 months (avg. 21 months) old mouse brain showing the clustering of cell types and progranulin positive nuclei as overlay (red dots). The images show each one representative mouse per genotype. The analysis included* n* = 2 Grn-flfl, n = 3 NesGrn KOBG and n = 3 PGRN KO mice. In Grn-flfl mice, progranulin is expressed mostly in neurons, and in few microglia. In NesGrn KOBG, progranulin is restored in neurons but not microglia

PGRN-KO mice [32] (gift from Aihao Ding, Grn^tm1Aidi^, MGI:4421704) were crossed with Gt(ROSA)26Sor^tm1.1(Ubc−Grn)Ite^ (MGI: 6149573), referred to as Grn-flfl mice [7] and with Nestin-Cre mice (B6. Cg-Tg(Nes-cre)^1Kln/J^). To generate the Grn-flfl mice, a targeting vector consisting in the murine cDNA of progranulin, upstream ubiquitin promoter and a loxP flanked STOP codon (fl-STOP-fl) was inserted via homologous recombination into the Rosa26 locus, leading to the generation of Gt(ROSA)26Sor^tm1.1(Ubc−Grn)Ite^ mice (MGI: 6149573) [7]. Subsequently, the transgene expression of progranulin by Cre-recombinase was achieved by crossing Grn-flfl mice with Nestin Cre mice (B6.Cg-Tg(Nes-cre)^1Kln/J^), thereby removing the floxed STOP-codon. The resulting mice were then crossed with progranulin knockout mice (B6.CD1-*Grn*^tm1Ding^) referred to as PGRN KO mice [32], which had been generated by insertion of loxP sites flanking the promoter and the first four exons of the progranulin gene via homologous recombination. These floxed mice were crossed with pan-Cre deleter mice (CAG-cre) to generate the full knockout [32]. The breeding strategy is shown in Fig. 1A.

Triple heterozygous offspring were further crossed to homozygosity for the PGRN knockout allele (PGRN KO) and the loxP-STOP-loxP-mGrn allele (Grn-flfl) and to hemizygosity of Nestin-Cre (NesCre). Cre-positive mice exhibited exclusively neuronal PGRN expression. This line is referred to as NesGrn KOBG. The short name stands for Nestin driven *Grn* expression in *k*nock*o*ut *b*ack*g*round where the endogenous *Grn* was deleted. Cre-negative animals carry an inactive floxed allele and are therefore PGRN KO mice. A triple genotyping assay consisting of 6 probes was developed and is available from Transnetyx (Line: NesGrn KOBG). All mice were on a C57BL6 genetic background. Suppl. Table S1 provides an overview of sample sizes, ages and sex of mice used for behavioral and biological experiments.

Mice had free access to water and food and were kept in climate-controlled rooms with a 12 h light-dark cycle. The behavioural studies were approved by the local Ethics Committee for animal research (Darmstadt, Germany) under FK1096, FK1103, FK1100 and FU2080. The studies adhered to the guidelines of the Society of Laboratory Animals (GV-SOLAS) and were in line with the European and German regulations for animal research and the ARRIVE guideline.

### Primary microglia culture

A Miltenyi Biotec Adult Brain Dissociation Kit and Miltenyi CD11b (Microglia) MicroBeads were used to isolate microglia from adult brain. Briefly, mice were euthanized with CO_2_ and total blood withdrawal via cardiac puncture, and the brains were collected in HBSS buffer. After washing with HBSS buffer, each brain was sliced into smaller pieces and collected in gentle MACS C tubes containing a preheated enzyme mixture (buffer Z and enzyme P) at 37 °C. The MACS enzyme mix (buffer Y and enzyme A) was added to each tube, which was subsequently incubated on a gentleMACS Octo dissociator at 37 °C and 50 rpm for 30 min. DNase I was subsequently added, followed by further incubation for 10 min at 37 °C and 50 rpm. HBSS buffer was added, and the tubes were incubated on ice for 5 min, then centrifuged, and the supernatant was removed. After resuspension in HBSS buffer, the samples were filtered through a 70 μm smart strainer, and the smart strainer was washed with HBSS buffer. The cell suspension was pelleted, and the supernatant was discarded. The pellet was again resuspended in HBSS buffer and MACS debris removal solution. After brief incubation, another 4 ml of cold HBSS buffer was added, cells pelleted and the upper two phases were removed, followed by another centrifugation step. Finally, the pellet was resuspended in red blood cell removal solution and incubated at 4 °C for 10 min. Binding buffer (PB: 0.5% BSA in HBSS) was added, the cells were again pelleted, and the supernatant was removed. The cell pellet was then resuspended in PB buffer, 15 µl of MACS CD11b magnetic microbeads were added, mixed, and the mixture was incubated at 4 °C for 15 min and then pelleted. The supernatant was removed, and the pellet was resuspended in PB buffer.

MiniMACS MS columns were prepared with 500 µl of PB buffer and mounted on a magnetic stand (Miltenyi). The cell suspension was added on top of the columns, and the columns were washed 3x with PB buffer. The flowthrough, containing non-microglial cells, mainly neurons and astrocytes, was collected and stored at −80 °C for later analysis. To elute microglia, 200 µl of PB buffer was added onto the columns and, after flow-through, another 800 µl PB buffer were pushed through the columns with the plunger. The collected cells were pelleted, and the supernatant was removed. The pellet was resuspended in 37 °C preheated medium (DMEM/F12-GlutaMax™ (200 µM) medium (Gibco) supplemented with 10% FCS and 1% PenStrep). The cells were counted in a Neubauer counting chamber, and approximately 50,000 cells were seeded into one well of an 8-well culture slide or were used for RNA extraction and subsequent quantitative rtPCR analysis.

For isolation of microglia from neonatal mouse brains (P1–P3) a standard procedure using enzymatic dissociation followed by mechanical shaking was used. After euthanasia, brains were dissected and meninges removed. Tissue was minced and digested with 0.05% trypsin-EDTA and DNase I at 37 °C for 15 min, then triturated to form a single-cell suspension and filtered through a 70 μm strainer.

Cells were centrifuged and resuspended in DMEM with 10% FBS and 1% PenStrep, then plated in poly-L-lysine (0.01% PLL) coated T75 culture flasks. Cultures were maintained for 7–9 days with half exchange of the medium every 3–4 days, allowing astrocytes to form a monolayer while microglia remained loosely attached. Floating microglia were collected. To isolate microglia, 12 mM lidocaine was added, and flasks were shaken until microglia lifted off. The supernatant was collected, centrifuged, and microglia were harvested or replated onto coated surfaces for further studies.

Primary DRG neurons of adult mice were prepared and cultured as described previously [7, 33, 34].

### Gene expression analysis using quantitative PCR

For *Grn* gene expression analysis in primary neural and glial cells and brain tissue RNA was isolated from primary microglia and neurons/astrocytes obtained during the isolation protocol. The Qiagen All Prep DNA/RNA/Protein Isolation Kit and the Qiagen RNA Isolation Kit were used according to the manufacturer’s instructions. The amount of isolated RNA and purity were measured on a Nanodrop spectrophotometer, and 180–200 ng of RNA and the Verso cDNA Synthesis Kit (Thermo Scientific) were used to generate cDNA via reverse transcription according to the manufacturer’s instructions. RT‒qPCR was conducted by using ORA™ SEE qPCR Green ROX (highQu) in duplicate on a QuantStudio™ 5 System (Thermo Fisher Scientific). Absolute values of *Grn* gene expression were normalized to the reference peptidylprolyl isomerase A (*Ppia*) and glyceraldehyde 3-phosphate dehydrogenase (*Gapdh*) values. Oligonucleotide sequences, amplicon sizes, annealing temperatures, and NCBI reference sequence numbers are provided in the Supplementary Material (Suppl. Table S4).

### Mice tissue collection: brain and plasma

Mice were euthanized with carbon dioxide and blood withdrawal by cardiac puncture, whereby blood was collected into K3^+^ EDTA tubes, centrifugated at 1300 g for 5 min, and plasma was transferred to a fresh tube and snap frozen on dry ice or in liquid nitrogen. The brain was dissected for lipidomic and transcriptomic analyses. Cerebellum and olfactory bulb were removed, and the brain was cut sagittal. Left and right halves were weighed with precision scales and snap frozen on dry ice. Samples were stored at −80 °C until analysis.

### Brain tissue processing and immunofluorescence analysis

Mice were terminally anesthetized with carbon dioxide and ketamine and transcardially perfused with cold phosphate buffered saline (PBS) followed by 2.25% paraformaldehyde (PFA) for fixation. Tissues were excised, postfixed in 2.25% PFA for 2 h, cryoprotected overnight in 20% sucrose at 4 °C, embedded in small tissue molds in cryo-medium and cut on a cryotome (12 μm). Slides were air-dried and stored at − 80 °C. Immunofluorescence staining was performed with air-dried cryosections. After washing in 1x PBS, sections were blocked in 0.5% Triton X-100/5% BSA/PBS at room temperature (RT) for 120 min and incubated in primary antibody solution (IBA1) solution consisting of 0.1% Triton X-100 and 1% BSA overnight at 4 °C. After washing, sections were incubated in secondary antibody for 2 h at RT. The steps were repeated for the second primary/secondary antibody pair (GFAP). Finally, nuclei were counterstained with DAPI for 10 min at RT, washed again and mounted with Aqua-Poly/Mount (Polysciences). A similar workflow was used for the staining of synapses using the primary antibodies PSD95 and SV2. Antibodies are listed in Suppl. Table S5.

### Golgi-Cox staining of brain tissue and quantification of spine density

The Golgi-Cox staining was performed with an FD Rapid GolgiStain™ Kit from FD NeuroTechnologies according to the manufacturer’s instructions. Mice were euthanized with carbon dioxide and transcardially perfused with cold 1x PBS followed by 2.25% paraformaldehyde (PFA) for fixation, and the brain was excised. A frontal brain slice of aproximately 5 mm thickness (Bregma + 0.5–2.5) was cut and submerged in 4 ml of the impregnation solution, which was exchanged with fresh solution after 24 h. The tubes were gently swirled and kept at room temperature (RT) for 14 days, protected from light. The brain tissue was then transferred into 4 ml Solution C and kept at RT in the dark for another 72 h during which Solution C was 1x refreshed. Subsequently, the brain tissue was embedded in 2% low-melting agarose and sectioned on a vibratome into 100 μm thick slices.

The sections were collected on superfrost microscope slides and allowed to air dry for 72 h at RT protected from light. Before staining, the sections were washed 2 × 4 min in MilliQ water and then placed for 10 min into the staining solution, which was freshly prepared by mixing 1 part Solution D with 1 part Solution E and 2 parts MilliQ water. Finally, the sections were washed for 2 × 4 min in Milli-Q water and then dehydrated in 50, 75 and 95% ethanol, each for 4 min, and then 100% ethanol 4 × 4 min. The slides were cleared 3 × 4 min in xylene, before embedding in Pertex mounting medium. The slides were dried overnight at 37 °C and stored at RT, protected from light. The BioRevo BZ9000 Keyence microscope; (RRID: SCR_015486) with 40x objective lens was used for imaging and image capture of the frontal cortex layer 2–3. For each image, one or two dendrites with a length of 30 ± 1 μm were analysed to obtain the spine density, which was done with FIJI ImageJ. Spine counts were normalized per 10 μm dendrite length.

### Image acquisition and analysis

Immunofluorescence images were captured using fluorescence microscopy (BZ-X800, Keyence) or confocal scanning microscopy (Leica Stellaris 8, RRID: SCR_024660). FIJI ImageJ (ImageJ, RRID: SCR_003070) was used for quantitative analysis of the immunoreactive (IR) area relative to the area of the region of interest (ROI) with adequate threshold setting using the particle analyzer plugin of ImageJ. The ROI was identical in size for all mice/sections. Four images/sections of each three mice were analyzed per region (motor cortex, hippocampus, thalamus, temporal cortex), resulting in 12 data points per genotype per region.

### Immunofluorescence analysis of primary microglia

Microglia were immune stained with anti-IBA1, and subsequent anti-mouse Alexa Fluor 488, and DAPI as nuclear counter stain. Microglia images were captured on a BioRevo BZ9000 Keyence microscope (RRID: SCR_015486).

For quantification, images were converted into 8bit binary images using the trainable weka segmentation tool in FIJI ImageJ and skeletonized. Subsequently, microglia morphometry was assessed by using the Fractal Generator (FracLac) plugin in ImageJ. The following parameters were obtained: (1) total foreground pixel within the ROI describing the size of the cell area (CA) including branches, (2) diameter of the surrounding circle, (3) area of a hull polygon connecting the tips of the outmost branches, (4) pixel density, defined as the ratio of the cell area (foreground pixel) to the area of hull polygon, (5) length-to-width ratio of the hull polygon, (6) counts of proximal branches, (7) counts of branch tips, (8) counts of junctions, (9) counts of triple junctions, (10) total lengths of branches, (11) ramification factor which is the ratio of proximal branches to soma size, (12) fractal dimension (FracD), which is an index describing the degree to which a complex structure fills out a graphic area and was calculated by a box-counting algorithm, (13) circularity, calculated according to the formula: circularity = 4π × cell area/CP^2^ where the cell perimeter (CP) is compared to that of a circle.

Multiple cells were analyzed from each image and were obtained from cultures of each three mice per genotype. For multivariate analyses, measures for the parameters were log2-transformed because the data were mostly log-distributed, except for fractal dimension and pixel density which were used as linear data. Transformed data were subjected to supervised Canonical Discrimination and Random Forest analysis to assess if/how complex microglia morphology predicted the genotype.

Data for individual parameters were submitted to Brown-Forsythe and Welch ANOVA and subsequent Dunnett’s T3 multiple comparisons test.

### RNA sequencing

Total RNA was extracted from fresh frozen brain tissue, which included cortex and subcortical structures. Briefly, total RNA was purified using an RNAeasy plus micro kit (Qiagen), and the quantity and quality assessment was checked by Qubit Flex and RNA 6000 Nano chip on Agilent’s bioanalyzer, respectively. Next Generation paired-end mRNAseq was performed at Novogene (Cambridge, UK) on an Illumina NovaSeq 6000 platform. Sample quality was evaluated using demultiplexed fastq.gz files. Sequenced reads were subjected to adapter trimming and processing via CLC Genomics workbench (Qiagen, v22) using standard setting for sequence alignment. Sequence reads were annotated according to the mouse genome mm10 assembly. Results were visualised using CLC expression browser, encompassing the number of mapped reads, target length, source length and position, strand, genes and gene IDs. Read counts were normalized using the EdgeR algorithm providing the trimmed mean of M-values, TMM (in CLC called CPM). TMM reads were Log2 transformed.

Differential gene expression was assessed using ANOVA-like methods and t-tests and fold change in CLC genomic workbench. Low expression genes were filtered out. The P value was set at 0.05 and adjusted according to the False Discovery Rate (FDR). Hierarchical clustering with Euclidean distance metrics was used to assess gene expression patterns. Results were displayed as heat maps with dendrograms. Genes were ranked according to P and q value, fold change and abundance. Ranked genes were submitted to gene ontology enrichment analysis using DAVID (Database for Annotation, Visualization and Integrated Discovery [35]) and the web tool Gorilla (https://cbl-gorilla.cs.technion.ac.il/). The RNAseq data have been deposited at the GEO database with the provisional accession number GSE273083.

### Single nucleus RNA sequencing

Single nucleus mRNA sequencing (snRNA-seq) was performed to profile gene expression from isolated nuclei derived from brain tissue samples of 20–23 month old mice (*n* = 2 Grn-flfl, *n* = 3 NesGrn KOBG, *n* = 3 PGRN KO). Nuclei isolation, library preparation and sequencing was performed by Novogene.

Tissues were dissociated to release intact nuclei while minimizing cytoplasmic contamination. Nuclei were purified using a combination of mechanical disruption and filtration, followed by quality assessment via microscopy and automated cell counters to ensure integrity and appropriate concentration.

Library preparation was carried out using the 10x Genomics Chromium Single Cell 3’ Gene Expression v3.1 platform, optimized for nuclear RNA. Individual nuclei were encapsulated into nanoliter-scale Gel Bead-In-Emulsions (GEMs), each containing barcoded oligonucleotides. Within each GEM, reverse transcription was performed to capture polyadenylated nuclear transcripts, incorporating unique molecular identifiers (UMIs) and cell-specific barcodes. After breaking the emulsions, cDNA was purified and amplified. The resulting cDNA was fragmented, end-repaired, A-tailed, and ligated with sequencing adapters, followed by PCR enrichment to generate the final libraries.

Library quality was assessed using Qubit fluorometry for concentration, Agilent Bioanalyzer for fragment size distribution, and qPCR for quantification of effective library concentration. Sequencing was performed by Novogene using the Illumina NovaSeq platform with paired-end reads —typically 28 bp for Read 1 (capturing barcodes and UMIs) and 91 bp for Read 2 (capturing transcript sequences). The sequencing depth was targeted at approximately 20,000 reads per nucleus, and 5,000–10,000 nuclei were sequenced per sample.

Raw sequencing data were stored in fastq files and processed using the Cell Ranger pipeline from 10x Genomics for each sample separately. Reads were aligned to the reference genome (Mus musculus genome assembly GRCm39 (mm39)) using STAR, and gene-barcode matrices in HDF5 file format were generated after correcting barcodes and UMIs. Downstream analysis was conducted using CLC Genomics workbench ver. 22.01 using the raw HDF5 files as input, which contain barcode-level UMI counts for each gene. The HDF5 matrix was imported into CLC Genomics Workbench using the Single Cell RNA-Seq plugin, which supports direct parsing of 10x Genomics output. Upon import, the tool automatically extracts gene annotations, cell barcodes, and UMI counts, generating a digital gene expression matrix suitable for further analysis.

Initial quality control was performed to filter out low-quality cells. Cells with fewer than 200 detected genes, high mitochondrial gene content (> 50%), or abnormally high UMI counts (potential doublets) were excluded. The remaining high-quality nuclei were normalized using counts-per-million (CPM) followed by log-transformation. Highly variable genes were identified using variance-based selection, and principal component analysis (PCA) was applied to reduce dimensionality. The top principal components were used for clustering via graph-based algorithms, including Louvain modularity optimization, to identify transcriptionally distinct cell populations. For visualization, Uniform Manifold Approximation and Projection (UMAP) and t-distributed Stochastic Neighbor Embedding (t-SNE) were employed to project cells into two-dimensional space, enabling intuitive exploration of cellular heterogeneity. Cells were annotated based on the expression of marker genes and known cell-type signatures using the cell type prediction tool. Differential expression analysis was conducted between gene clusters and genotypes using non-parametric statistical tests (e.g., Wilcoxon rank-sum). Data are presented as UMAP, tSNE and dot plots.

### Untargeted lipidomic analyses

Mouse brain tissue samples were homogenized by adding ethanol: water (1:3, v/v, tissue concentration 0.02 µg/ml) using a Precellys 24-Dual tissue homogenizer coupled with a Cryolys cooling module (both Bertin Technologies, Montigny-le-Bretonneux, France) with 10 zirconium dioxide grinding balls (3*20s at 6500 g with 60 s breaks), operated at < 6 °C. Subsequently, 20 µl of the homogenate containing 1 mg of tissue were extracted using a liquid-liquid-extraction method.

Lipidomic analyses were conducted applying the same procedure as previously described [36]. Further protocol details for extraction and analyses are described in the supplementary methods (Excel file). Briefly, a methyl-tert-butyl-ether (MTBE) and methanol-based liquid-liquid extraction was used. For chromatographic separation, a Zorbax RRHD Eclipse Plus C8 1.8 μm 50 × 2.1 mm ID column (Agilent, Waldbronn, Germany) with a pre-column of the same type was used. The mobile phases were (A) 0.1% formic acid and 10 mM ammonium formate and (B) 0.1% formic acid in acetonitrile: isopropanol (2:3, v/v). Analysis was performed on an Orbitrap Exploris 480 with a Vanquish horizon UHPLC system (both Thermo Fisher Scientific, Dreieich, Germany). Data was acquired using Thermo Scientific XCalibur v4.4 (RRID: SCR_014593) and relative quantification was performed in Thermo Scientific TraceFinder 5.1 (RRID: SCR_023045). Full scan spectra were acquired from 180 to 1500 *m/z* for lipidomics at 120,000 mass resolution each for 0.6 s, and data dependent MS/MS spectra at 15,000 mass resolution in between. Pooled quality controls were prepared from the first ten tissue homogenates and replicates measured along the run to verify system performance.

For all lipid analyses, the area under the curve (AUC) divided by the AUC of the Internal standard (AUC/IS) was used for quantification and statistical analyses. AUC/IS were transformed to square root of the AUCs to adjust skewed distribution. For multivariate analyses sqrt AUC/IS were scaled to have a common average and standard deviation of 1 (autoscaling in MetaboAnalyst).

### IntelliCage behavior

Behavioural analyses were done with unbiased IntelliCages. The IntelliCage (TSE, Berlin Germany) consists of four operant corners, each with two water bottles, sensors, LEDs, and doors that control the access to the water bottles. The system fits into a large cage (20 × 55 × 38 cm, Tecniplast, 2000P) and allows housing of 16 mice per cage. Four triangular red houses are placed in the center to serve as sleeping quarters and as stands to reach the food. The floor is covered with standard bedding. Mice are tagged with radiofrequency identification (RFID)-transponders, which are read with an RFID antenna integrated in the corner entrances. The corners give access to two holes with water bottles, which can be opened and closed by automated doors. Mice must make nosepokes (NP) i.e. peak through a light barrier to open the doors for water access. The IntelliCage is controlled by IntelliCage Plus software, which executes pre-programmed experimental tasks and schedules. The numbers and duration of corner visits, NP, and licks are recorded continuously without the need for handling of the mice during the recording times. Learning and memory can be supported by LEDs.

IntelliCage tasks address several different aspects of cognition as well as circadian rhythms and social interactions and were run sequentially. The tasks followed previously established protocols [37–39]. The IntelliCage experiments were done in young and old *female* mice to avoid fighting. Up to 16 mice were housed per cage. The schedules of the tasks for young and old were comparable, and details are presented in Suppl. Table 2. Mice were adapted to the cages for 1–2 weeks with free access to every corner, with all doors open, and water and food ad libitum. This free adaptation (FA) was followed by “nosepoke adaptation” (NP) for 1–2 weeks in which the doors were closed. The first NP of the visit opened the door for 5 s. To drink more, a mouse has to leave the corner and start a new visit. In the place preference learning (PPL1) task mice learned to prefer a specific corner in which an NP opened the door to get water access. Each 4 mice were assigned to one corner. After conditioning to the corner for 1–2 weeks, the rewarding corner was switched to the opposite side (PPL reversal learning (PPL2r) for another 1–2 weeks, and then again to a different long-side distant corner (PPL3) Some tasks were done with day patterns meaning that the learning module was only active during the night (young mouse cohort) or 2 × 3 h daily (old cohort). Outside of the module-active times, the doors remained closed.

### Statistics

Group data are presented as mean ± SD, mean ± sem for behavioral data, or median ± IQR as specified in the respective figure legends. Data were analyzed with SPSS 29 (RRID: SCR 016479) and GraphPad Prism 9 or 10 (RRID: SCR_002798), Origin Pro 2024 (RRID: SCR_014212), and MetaboAnalyst 5.0 (RRID: SCR_016723) (https://www.metaboanalyst.ca) [40]. Bioinformatic analyses of “omic” data (RNAseq, lipidomic, metabolomic) and microglia morphology are explained in the respective paragraphs. Mass spectrometry area under the curves (AUC) of lipidomic analyses were transformed to square root values to adjust skewed distributions. For multivariate analyses and presentations as heatmaps, data were normalized to have a common average and variance of 1 (Z-scores= (x-x̄)/SD). For testing the null-hypothesis that groups were identical, two groups were submitted to 2-way analysis of variance (ANOVA) using e.g., the factors “feature” (e.g. lipid, gene expression, behavioral feature) and ‘group’ representing the mouse lines. In case of significant differences, groups were mutually compared using post hoc t-tests according to Šidák or false discovery rate (FDR) or Dunnett versus the Grn-flfl control group. The meaning of asterisks in figures is explained in legends. Partial Least Square discrimination analyses (PLS-DA) and Random Forest supervised learning algorithms were used for lipidomic and transcriptomic data to assess the prediction of group membership and classification according to their importance. Volcano plots were used to assess fold differences of genes or lipids versus the negative logarithm (Log10) of the t-test P value according to standard procedures. Hierarchical clustering according to Euclidean distance metrics and Ward method were used to visualize lipidomic and transcriptomic data as heatmaps with dendrograms. The selection of top 100 or top 50 regulated genes or lipids was based on ANOVA *P*-values. ANOVA-simultaneous component analysis (ASCA) [41] and Canonical Discrimination Analysis were used for analysis of multiple behavioral features in sequential tasks. ASCA is a combination of ANOVA and PCA plus feature extraction method for multivariate data to model two major components and their interaction, which were “genotype” and “time/task”. The feature extraction is based on “leverage”, which is a measure of the importance of a feature’s contribution to the multivariate fitted ASCA-model, and the squared prediction error (SPE), which is an evaluation of the goodness of fit of the model to a particular feature.

## Results

### Progranulin expression is restored in neurons of NesGrn KOBG mice

The aim was to create mice which have progranulin selectively restored in neurons in a full progranulin knockout background (NesGrn KOBG). The breeding strategy is shown in Fig. 1A. rtPCR studies of *Grn* mRNA expression in various types of neuronal tissue, isolated neurons and glia of newborn up to old mice (Fig. 1B-E) show that the strategy was mostly successful. In newborn NesGrn KOBG, progranulin expression in neurons was fully restored (1B, D) owing to the high activity of the Nestin promoter during development. Expression in postnatal brain and DRG neurons even exceeded the expression in the floxed control mice, but only temporarily. RNAseq studies in old mice showed that *Grn* mRNA expression in NesGrn KOBG brain tissue was about one third of the level of the Grn-flfl controls (1 C) reflecting that *Grn* in the brain is contributed by neurons and microglia and that Nestin-driven Cre activity does not provide full transgene activation at this age. Progranulin protein in brain homogenates and plasma (1 F) was very low or not detectable in PGRN KO. It was also very low in plasma in NesGrn KOBG, where the source may be peripheral neurons or CNS derived extracellular vesicles. Progranulin protein in the brain was about half of the normal in both NesGrn KOBG and in PGRN het mice. Single nucleus RNAseq studies (Fig. 1G) show that progranulin is evenly distributed in different types of neurons and in microglial cells in the brain of Grn-flfl mice, with 250–265 *Grn* positive cells per mouse. In NesGrn KOBG, progranulin expression is restored in neurons without preference for specific types of neurons but not in microglia, overall 165–180 *Grn*-positive cells per mouse. In PGRN KO mice, there are few remaining *Grn*-positive barcodes (about 30) in neurons.

### Equal primary microglia morphology in NesGrn KOBG and full PGRN KO mice

**Fig. 2:**
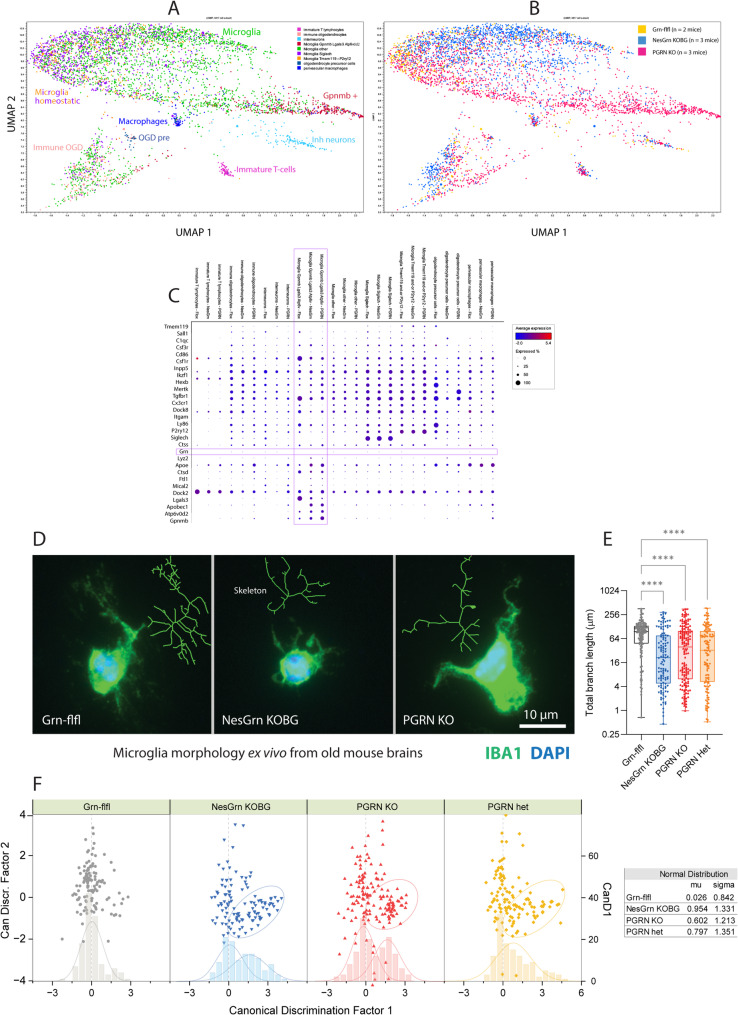
Microglia subtypes and morphology **A**, **B**: UMAP plots of snRNAseq analysis of microglia subtypes in old mouse brain in Grn-flfl, NesGrn KOBG and PGRN KO mice (12-24 months). In **A**, cells are colored by transcriptionally defined subtypes. In B, the cells are colored by genotype. The UMAP overview of all brain cell types is shown in Suppl. Figure S1.**C**: Dot plot analysis of microglia marker gene expression of subpopulations shown in **A** and **B**. The pink rectangle highlights a subpopulation of Gpnmb +, Atp6v0d2 + and Lgals3 + microglia which differed between genotypes. In progranulin deficient mice (NesGrn KOBG and PGRN KO) this population shows reduced expression of homeostatic microglia marker genes (Tmem119, P2ry12, Csf1r) but increase of Gpnmb, Atp6v0d2, Ctss, Lyz2, Apoe, Apobec and Hexb. **D**: Microglia morphology of primary microglia from old mouse brains (14-22 months), stained with anti-IBA1 and DAPI. Inserts in green show the skeleton of the respective cell, which was used for analysis. **E**: Quantitative analysis of the total branch lengths. Each scatter is one microglia cell. A total of 117-148 cells of 3-4 animals per genotype were analysed. The box is the interquartile range, the line is the median, and whiskers show minimum to maximum. Branch length data were submitted to Brown-Forsythe and Welch ANOVA and posthoc analysis using a correction of alpha according to Dunnett. P****<0.0001. **F**: For multivariate analyses of complex microglia morphology features (Supp. Fig. S1) data were submitted to Canonical Discrimination (CanDisc) analysis, and CanDisc scores for factor-1 and factor-2 are presented as XY scatter plots. The distribution of CanDisc-1 scores shows a biphasic distribution revealing “healthy” and “activated” microglia in progranulin deficient mice. The “activated” fraction is encircled

Single nucleus RNAseq studies (Fig. 1G) show a subpopulation of highly *Grn*-positive microglia in Grn-flfl mice which are not present in either knockout. Detailed analysis of microglial subpopulations (Fig. 2A, B, C) reveals a subpopulation of *Gpnmb*-positive microglia in PGRN KO brains, which are neither detectable in Grn-flfl mice nor in NesGrn KOBG mice. These cells are mostly positive for the lysosomal marker Lgals3 and/or Atp6v0d2, Apobec1 and the phagocytosis receptor Axl. Microglia with a similar phagocyte signature have been described a key pathologic microglial subpopulation accounting for neuronal loss in Prion-disease (10.1101/2025.02.11.637592). Dot plot analysis (Fig. 2C) shows that genotype dependent differences are prominent in this *Gpnmb*-positive subpopulation whereas homeostatic microglia marker expression is similar (e.g. *Siglech*, *Tmem119*, *P2ry12*). Mostly, up- or downregulations in NesGrn KOBG and PGRN KO are similar, but stronger in PGRN KO.

Despite the successful neuronal *Grn* restoration in NesGrn KOBG mice (Fig. 1G), which was associated with suppression of a highly phagocytic microglia subpopulation (Fig. 2A-C), ex vivo primary microglia of aged to old NesGrn KOBG mice showed an activated morphologic phenotype, which was indistinguishable from that of PGRN KO mice (Fig. 2D, 2E, Suppl. Fig. S2). There was no difference in any of the morphologic features between NesGrn KOBG and PGRN KO microglia. Canonical discrimination analysis of multiple morphologic parameters revealed a biphasic distribution for factor-1 scores in all progranulin knockout lines including heterozygous PGRN het mice (Fig. 2F) representing the normal and pathologically activated microglia subpopulations. It has to be considered that the *Gpnmb*-positive subpopulation is relative small in numbers and may not occur as a distinct subpopulation in ex vivo morphology studies. 

### Equal microgliosis and astrogliosis but partially restored spines in NesGrn KOBG

**Fig. 3:**
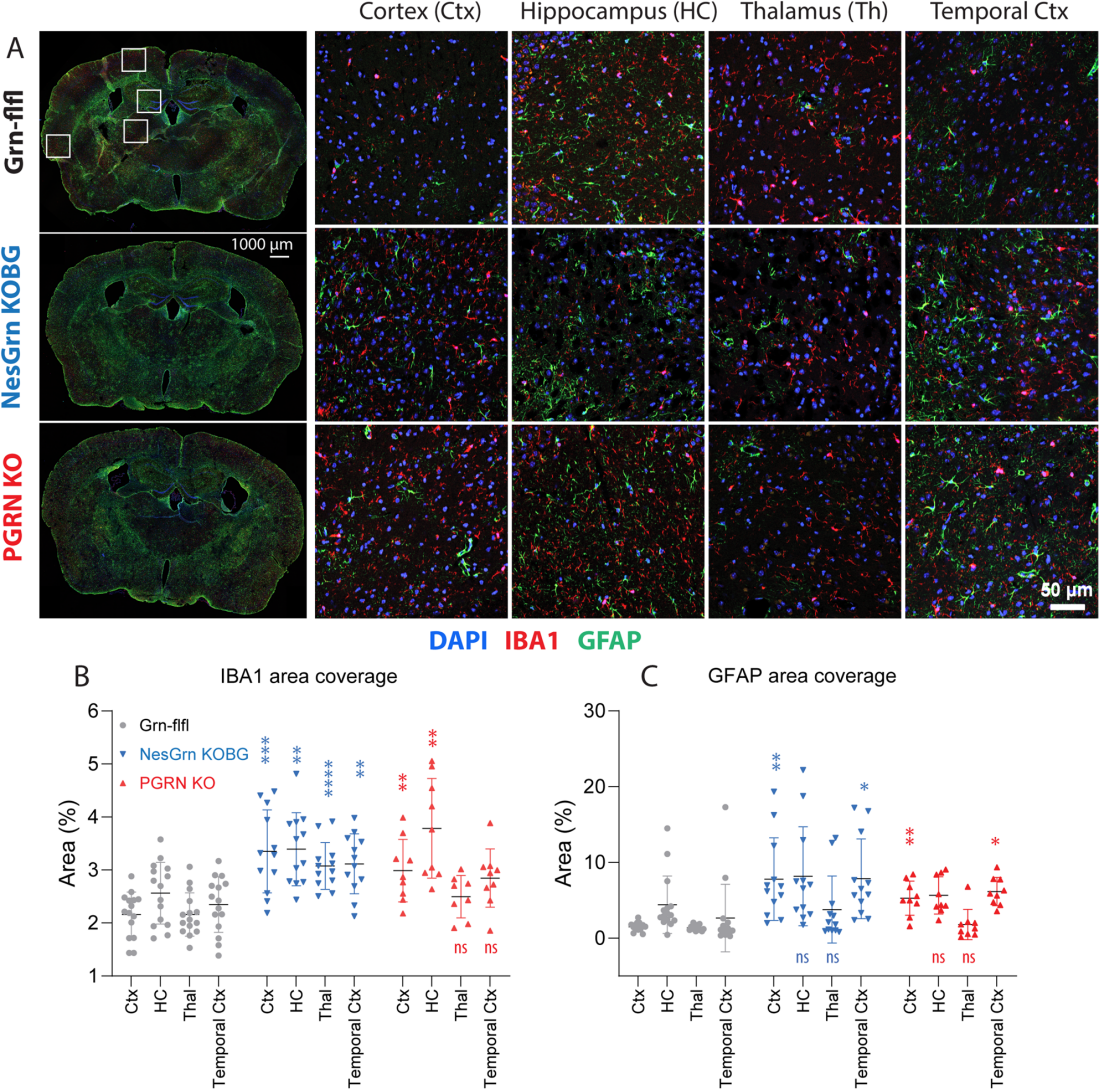
Microgliosis and astrogliosis in cortex, hippocampus and thalamus in progranulin deficient mice **A: **Exemplary immunofluorescent images of IBA1 immunoreactive microglia and of GFAP immunoreactive astrocytes in the cortex, hippocampus, thalamus and temporal cortex of Grn-flfl control mice, of NesGrn KOBG mice and of full PGRN KO mice (9-22 months). DAPI is used as nuclear counterstain. The left panel shows the overview, and the squares indicate which regions were captured at higher magnification. Microglia CD11b and CD68 activity marker expression is shown in Suppl. Fig. S3. More detailed analyses of Iba1/GFAP and of CD11b/CD68 of n = 3-5 mice is shown in Suppl. Histology.**B, C:** For quantitative analysis, images were converted to binary images using auto-threshold in FIJI ImageJ, and the relative area covered by specific immunofluorescence for either IBA1 or GFAP was used for statistical comparison. The scatters show results per image of n = 3-5 mice per genotype. The line is the average, and whiskers show the standard deviation. Data were submitted to 2-way ANOVA for “brain region” by “genotype”. Each region was then compared to the respective region of the Grn-flfl mice using an adjustment of alpha according to Dunnett. Asterisks reveal significant differences versus Grn-flfl. P*<0.05, **<0.01, ***<0.001,****<0.0001

Microglial progranulin deficiency leads to profound microgliosis and astrogliosis. Immunofluorescent studies in old mice using IBA1 for microglia and GFAP for astrocytes confirmed the expected gliosis in PGRN KO in the cortex and hippocampus (Fig. 3A-C, Suppl. Histology). Further immunofluorescence studies of the microglia activity markers, CD68 and CD11b [42], show a strong increase, particularly of CD68 at all sites (Suppl. Fig. S3; Suppl. Histology). CD11b was similar to Iba1. Both are general microglia marker proteins. CD68, also known as macrosialin or LAMP4, is a lysosome-associated membrane glycoprotein, which is strongly upregulated in activated microglia [43, 44]. Microgliosis and astrogliosis were not attenuated in NesGrn KOBG mice. There was no statistically significant difference of site-specific microgliosis in NesGrn KOBG versus PGRN KO mice.

**Fig. 4:**
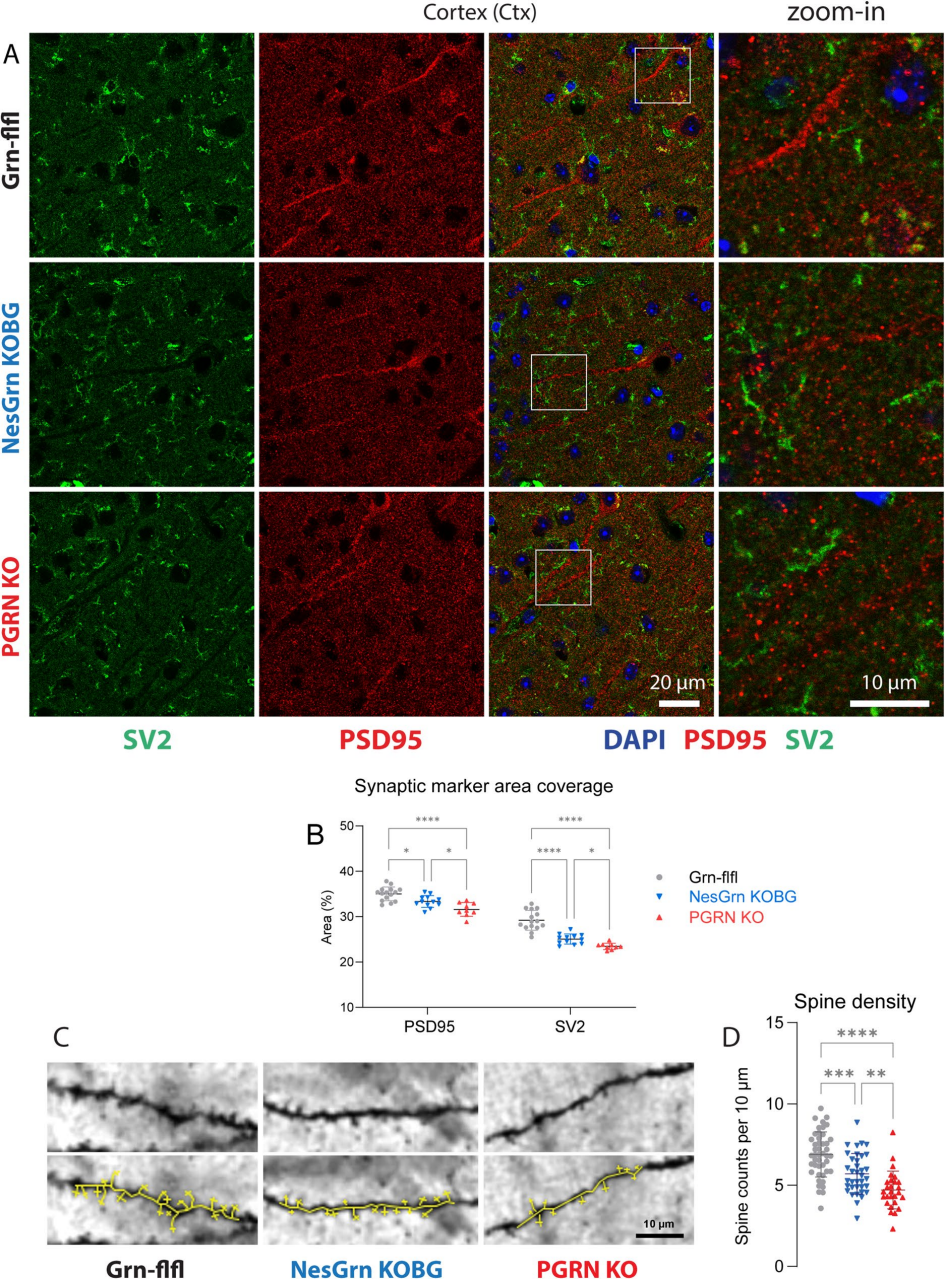
Synapses in progranulin deficient mice **A: **Exemplary immunofluorescent images of the postsynaptic marker PSD95 and presynaptic SV2 protein expression in the cortex of Grn-flfl control mice, of NesGrn KOBG mice and of full PGRN KO mice (9-22 months). Corresponding images of the hippocampus are shown in Suppl. Fig. S4.**B:** Quantitative analyses were done as in Fig.3. The scatters show results per image of n = 3-5 mice per genotype. The line is the average, and whiskers show the standard deviation. Data were submitted to 2-way ANOVA for “brain region” by “genotype” and subsequent posthoc analysis using an adjustment of alpha according to Tukey. Asterisks reveal significant differences. P*<0.05, ****<0.0001 **C:** Exemplary images of Golgi Cox staining of dendritic spines in the frontal cortex of Grn-flfl, NesGrn KOBG and full PGRN KO mice (9-22 months). The yellow markers in the bottom row highlight the spines which were counted. **D:** Spine counts were normalized per 10 µm of the dendrite length representing the “spine density”. Each scatter shows the density of spines of one dendrite. At least 9 dendrites were quantified per mouse of n = 3-5 mice per genotype. Data were submitted to one-way ANOVA and subsequent posthoc comparison according to Tukey. P**<0.01, ***<0.001, ****<0.0001

Progranulin deficient microglia are known to strongly upregulate complement factors and excessively prune synapses [45]. It is not clear if microglia receive more or stronger eat-me signals from progranulin deficient neurons or attack axons or dendrites without appropriate neuronal signal following an inherent aggressive “autopilot”. Immunofluorescence studies of synaptic structures in cortex (Fig. 4) and in the hippocampus (Suppl. Fig. S4) showed that PSD95 immunoreactive postsynaptic structures were significantly reduced both in PGRN KO and in NesGrn KOBG mice, but the loss was significantly stronger in PGRN KO than NesGrn KOBG mice. Synaptic vesicle glycoprotein 2 (SV2) immunoreactive presynaptic structures were reduced in the cortex but not in the hippocampus. Again, SV2 loss in the cortex was stronger in PGRN KO than NesGrn KOBG mice.

Golgi-Cox staining (Fig. 4C and D) showed a strong spine loss in PGRN KO mice which was partly attenuated in NesGrn KOBG mice suggesting that more synapses were preserved in NesGrn KOBG despite equal inflammation. Together with our snRNAseq studies, we infer that the cascade of the pathology starts with excessive posting of eat-me-signal by endangered neurons resulting in a transformation of microglia into Gpnmb-positive phagocytic microglia which then excessively prune synaptic spines and further attack intact synapses and thereby perpetuate the damage.

### Equally upregulated immune genes in NesGrn KOBG and PGRN KO mice

**Fig. 5:**
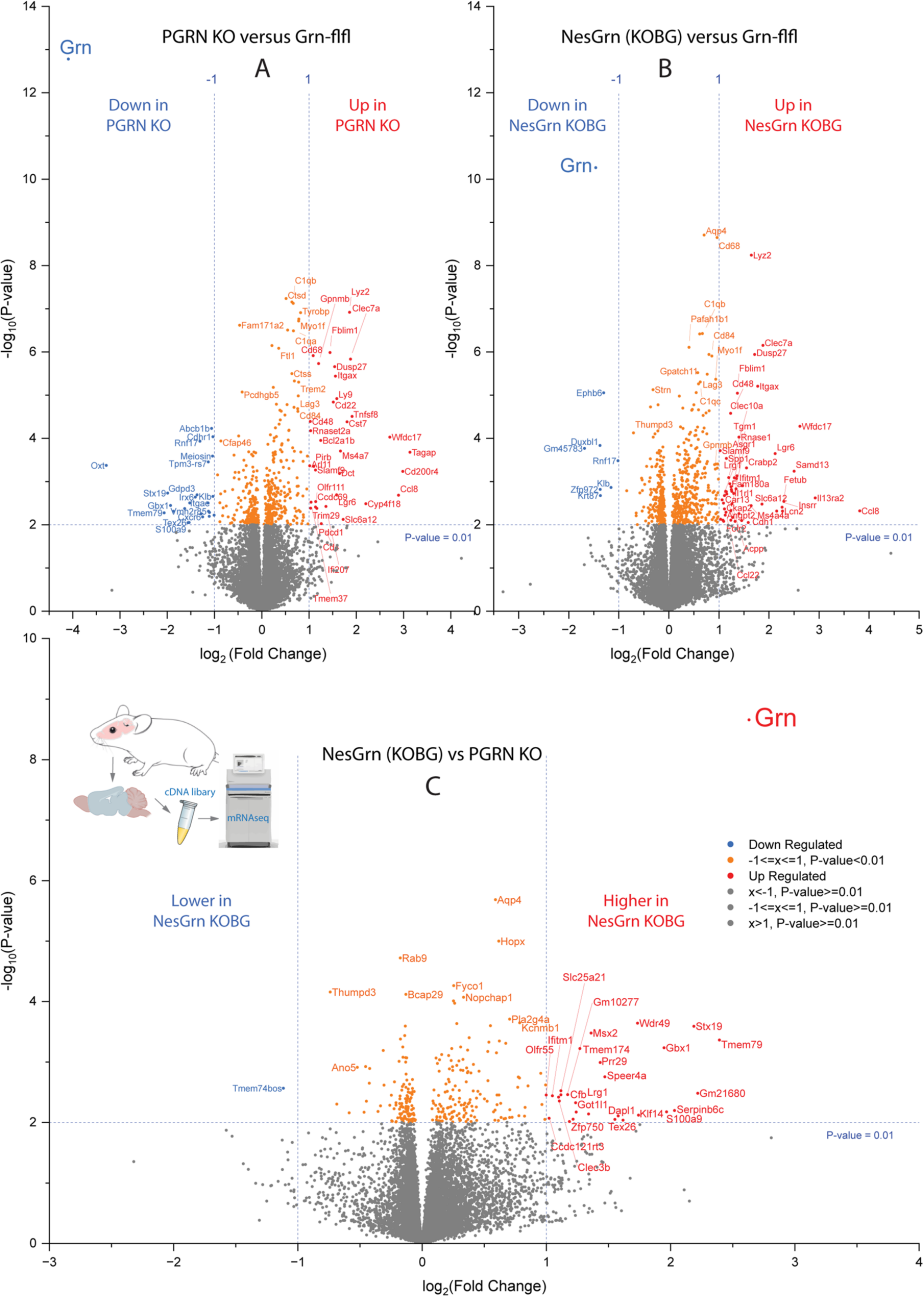
Brain transcriptome in progranulin deficient and Grn-flfl control mouse mice **A**: Volcano plot of RNAseq of old PGRN KO versus Grn-flfl control mice (13-15 months). The x-axis shows the Log2(Fold change) of normalized counts (CPM). The y-axis shows the negative logarithm of the t-test P-value. The data are from n = 8 mice per group. Upregulated genes are shown in red, downregulated in blue. **B**: Volcano plot of RNAseq of old NesGrn KOBG versus Grn-flfl control mice (13-15 months). Graph settings and sample size as described under A. **C**: Volcano plot of RNAseq of old PGRN KO versus old NesGrn KOBG control mice. Graph settings and sample size as described under A

**Fig. 6:**
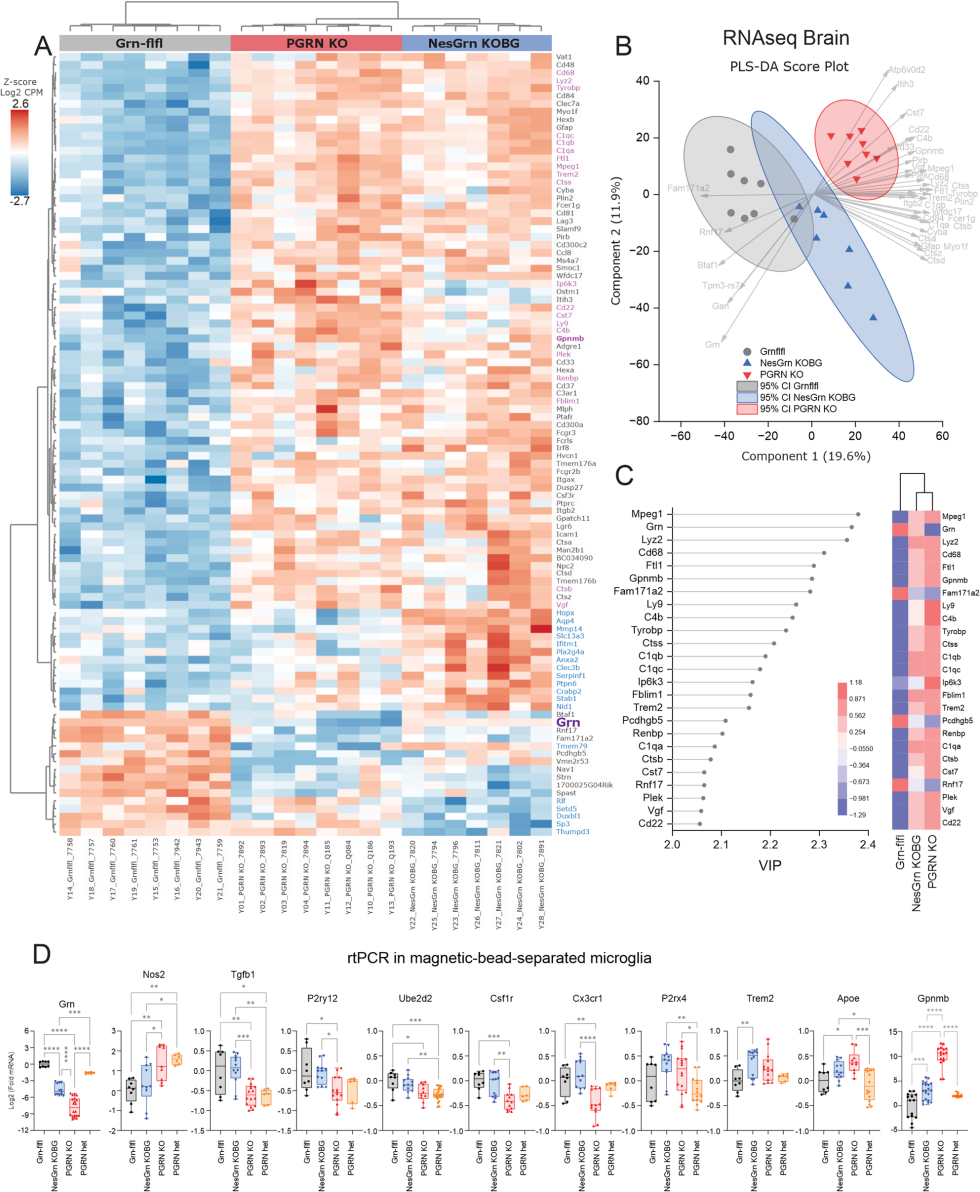
Differential gene expression of candidate genes in old mice brains **A**: Heatmap of RNAseq top 100 regulated genes, selected by ANOVA P values, and hierarchical clustering of genes and mice. The tree shows Euclidean distance metrics, Ward method. Gene names in blue indicate difference between PGRN KO and NesGrn KOBG. *Grn* itself is highlighted in violet and bold letters. Genes identified in the variable importance plot (Fig. 6C) are highlighted in pink. Mice as in Fig.5. **B**, **C**: Score plot and variable importance (VIP) plot of Partial Least Square discrimination analysis (PLS-DA) of RNAseq normalized counts. A total of 3178 ANOVA significant genes (raw P, non-FDR adjusted) were submitted to PLS-DA. The VIP ranking agrees well with the ranking according to ANOVA P values.**D**: rtPCR mRNA analysis of candidate genes of primary microglia which were obtained from *n *= 3-6 mice per genotype and split into two samples each (broad age range covered 3-17 months, matched pairs). The box is the interquartile range, the line is the median, and whiskers show minimum to maximum. Data were submitted to one-way ANOVA and subsequent posthoc t-tests. The asterisks show Šidák adjusted P values. P*<0.05, **<0.01,***<0.001, ****<0.0001

To assess if and how neuronal progranulin restoration was able to reduce the pathology caused by progranulin loss at the molecular level we used bulk mRNAseq with total RNA extracted from old mouse brain tissue. Volcano plots of differentially expressed genes show very similar results for PGRN KO versus Grn-flfl controls and NesGrn KOBG versus Grn-flfl controls except for progranulin itself (Fig. 5). The upregulated genes are almost exclusively immune genes, and the results agree with previous RNAseq studies in progranulin-knockout brain tissue [20, 27, 46–48]. Detailed analyses of the upregulated immune genes (Suppl. Fig. S5A, B) and cluster analysis of top upregulated genes (based on ANOVA statistics, Fig. 6A) showed that there was indeed almost no difference between PGRN KO and NesGrn KOBG mice. Most of the top upregulated genes are microglia-associated genes [49]. The leading candidate genes defining progranulin disease-associated-microglia (DAM) are similar but not equal to DAM RNA signatures in models of Alzheimer's Disease [50–52] and include *Lyz2*, *Mpeg1*, *Gpnmb*,*Tyrobp*, *Cd68*, some complement factors (C1q’s), some cathepsins (Cts’s),*Trem2*, *Hexa*, *Hexb*, *Lag3*, integrins and immunoglobulin receptors (Fig. 6, Suppl. Fig. S5A, B). They have all been described as microglia marker genes in mice [49]. Direct comparison of NesGrn KOBG versus PGRN KO in Volcano plots show higher expression of some neuronal and astroglial genes in NesGrn KOBG, and analysis of genes which are lost in PGRN KO but preserved in NesGrn KOBG revealed that the preserved genes are involved in axonal structures and dynamics (dynein complex) (Suppl. Fig. S5C-S5E, pathways S5F and S5G) such as dynein light chain and heavy chain subtypes (Dnali1, Dnah11, Dnah11), and axonemal central pair apparatus protein, Hydin and cilia associated proteins (e.g. Cfap44). Score plots of PLS-DA, which was run with all ANOVA significant genes (3178 genes based on non-corrected *P*-value), show three clusters, with NesGrn KOBG in the middle (Fig. 6B), which agrees with partial progranulin restoration. The most important genes obtained by PLS-DA (Fig. 6C, Variable Importance Plot) agree with ANOVA P-values of the most strongly regulated immune genes. Upregulation of a panel of selected microglia relevant genes in isolated primary microglia was confirmed by rtPCR analysis also including PGRN het mice (Fig. 6D). Homeostatic microglia marker genes including *P2ry12*,*Tgfb1*, *Csf1r* and *Cx3cr1* were downregulated in PGRN KO but not in NesGrn KOBG microglia. The rtPCR results suggest that progranulin in or from neurons kept some microglia in a resting state. These genes were not significantly regulated in bulk RNAseq studies, but normalized counts of *Tgfb1*, *Csf1r* and *Cx3cr1* were higher in NesGrn KOBG than PGRN KO mice. In snRNAseq data Fig. 2C), homeostatic microglia marker gene expression was similar in NesGrn KOBG and PGRN KO mice.

### Lower carnitines and increased phosphatidylserines in PGRN KO versus NesGrn KOBG mice

**Fig. 7:**
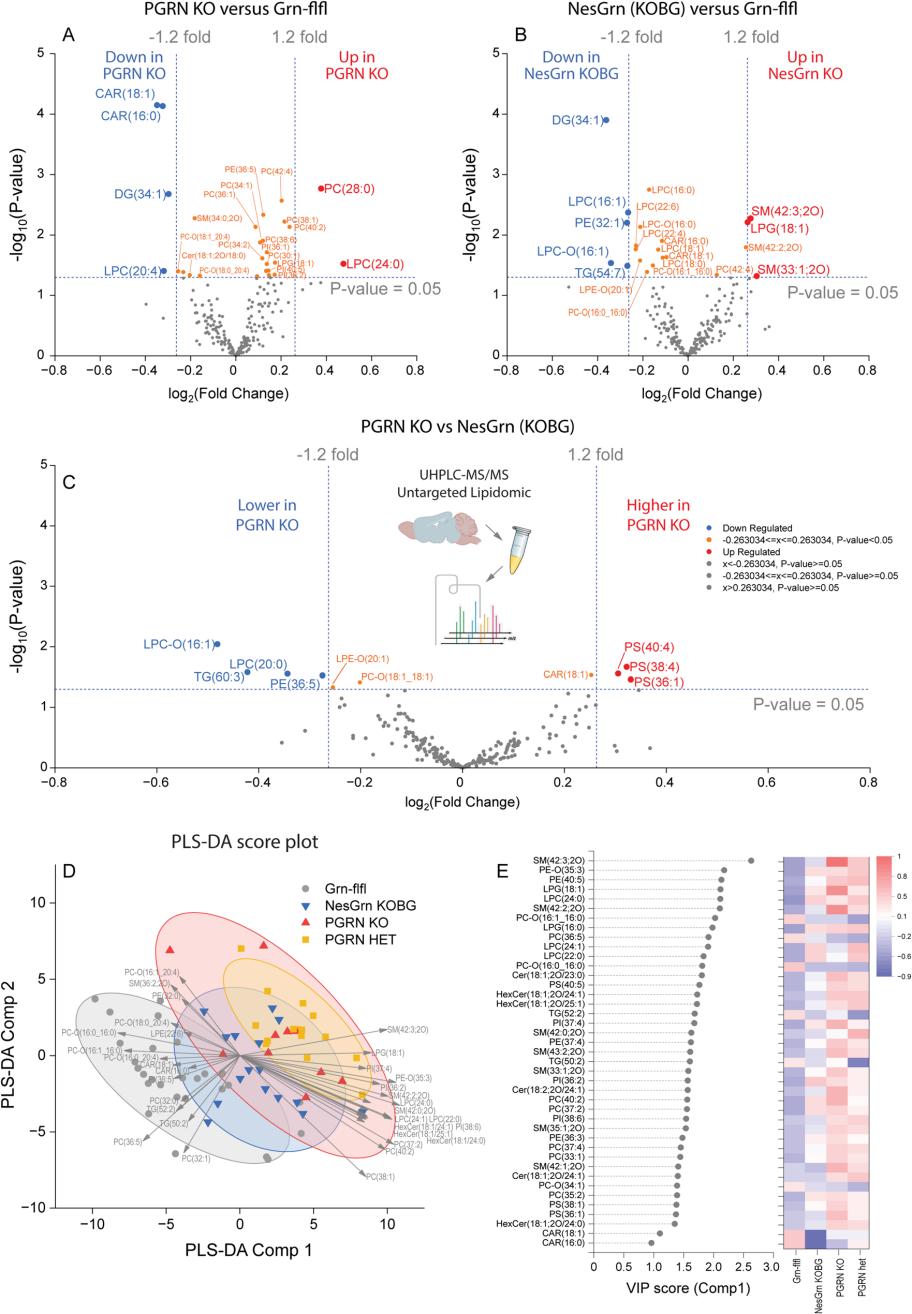
Untargeted lipidomic analysis in the brain **A, B, C**: Volcano plots showing lipid species in the brain of PGRN KO mice versus Grn-flfl control mice (A), NesGrn KOBG versus Grn-flfl control mice (B) and PGRN KO versus NesGrn KOBG mice (C). The data are from n = 27 Grn-flfl mice, n = 10 PGRN KO and n = 16 NesGrn KOBG mice (age 6 months and 13-15 months, matched). Lipids were analysed by untargeted UHPLC-MS/MS lipidomic screening. The X-axis shows the log2(Fold change), the Y-axis minus Log10 of the t-test P-value. Upregulated lipids are shown in red, downregulated in blue. **D, E:** Score biplot and variable importance (VIP) plot of Partial Least Square (PLS-DA) analysis of brain lipids analysed by UHPLC-MS/MS lipidomic screen. The VIP agrees with the ranking according to t-test P values. The arrows in D point to the loading to top regulated lipids. PGRN heterozygous (het) mice were included in some experiments. Abbreviations: CER, ceramides; CE, cholesterol ester; DG, diglycerides; FA, fatty acids; HexCer, hexosylceramides; LPC, lysophosphatidylcholines; LPE, lysophosphatidylethanolamines; LPG, lysophosphatidylglycerols; LPI, lysophosphatidylinositols; PC, phosphatidylcholines; PE, phosphatidylethanolamines; PG, phosphatidylglycerols; PI, phosphatidylinositols; SM, sphingomyelins; ST, sterols; TG, triglycerides; –O ether bound lipids

Previous histologic studies have shown that progranulin deficient microglia accumulate large amounts of neutral lipids [21, 53, 54] likely caused by pathologic scavenging and defective reutilization through lipophagy. Plin2, which is a marker of lipid droplets, was significantly upregulated (RNAseq Heatmap Fig. 6A). In addition, phosphatidylserine (PS) species exposed to the outer surface of membranes are strong eat-me signals. To get further insight into the progranulin-associated pathology of brain lipids we employed a lipidomic screening of old mouse brain tissue. The differential regulation of lipids is shown as Volcano plots (Fig. 7A-C) and PLS-DA score plot and Variable Importance plot (Fig. 7D, 7E). The PLS-DA loading is in Suppl. Fig. S6. There were only modest changes of lipidomic patterns in PGRN KO or NesGrn KOBG mice, and there were no consistent changes of multiple species of one class. Specifically, there was no accumulation of cholesterol or tri- or diglycerides which are indicators of brain injury or disease [55–57]. Long-chain carnitines (CAR16:0 and CAR18:1) were reduced in PGRN KO but not in NesGrn KOBG, suggesting a reduction of mitochondrial mass or higher consumption in PGRN KO. Carnitines transfer long chain fatty acids through the inner mitochondrial membrane to supply beta oxidation. Three PS species were increased in PGRN KO versus NesGrn KOBG which would agree with stronger presentation of eat-me signals in full knockouts leading to synaptic loss. PLS-DA score plots show broad overlap of all progranulin deficient lines including PGRN het mice.

### IntelliCage behavior shows age-dependent compulsive behavior in both KO lines

**Fig. 8:**
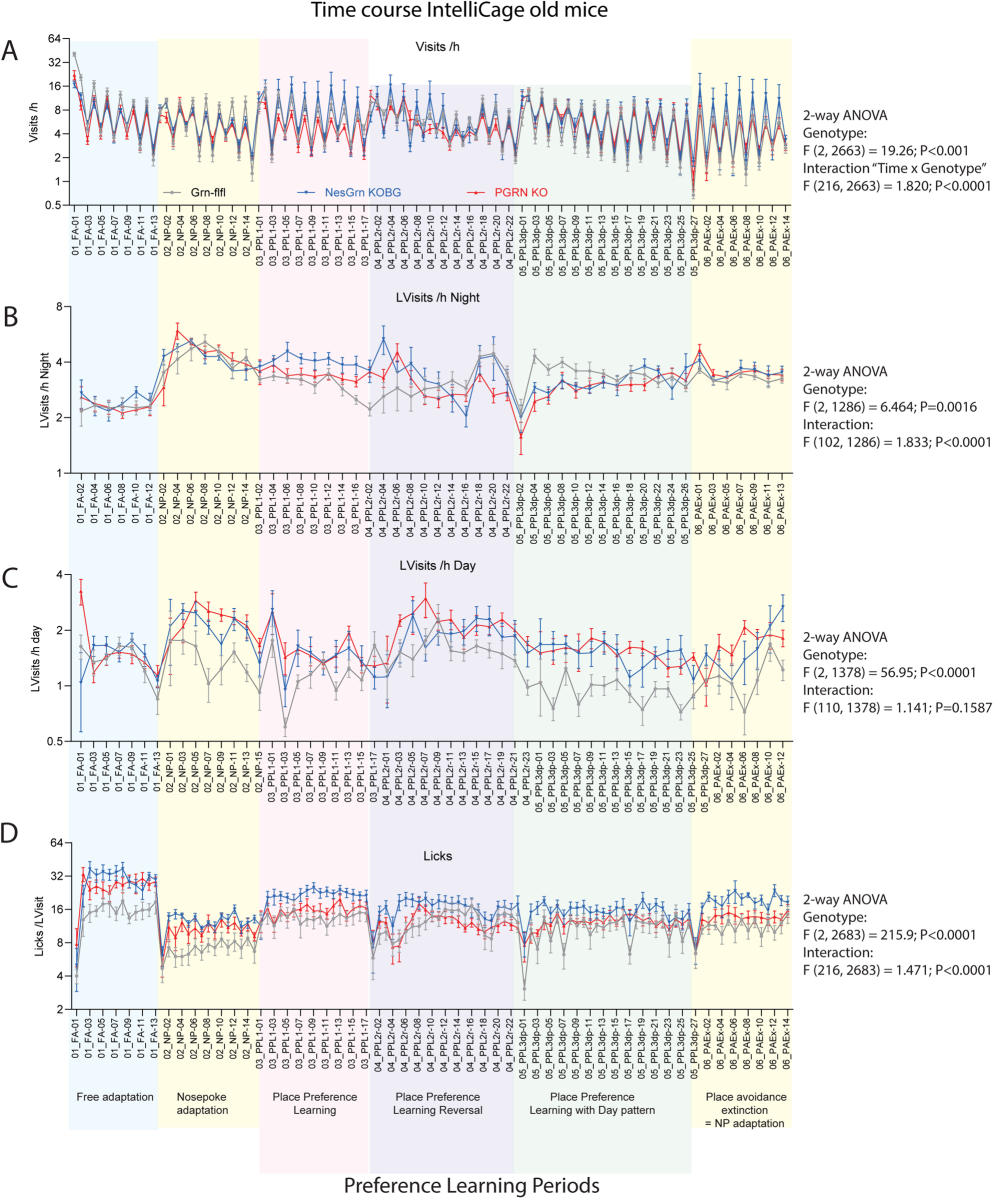
Longitudinal behaviour of old Grn-flfl, PGRN KO and NesGrn KOBG in IntelliCages **A** Time course of corner visits per hour (Visits /h) during different tasks in IntelliCages. **B** Time course of corner visits with licks per hour at night (LVisits /h Night). **C** Time course of corner visits with licks per hour at day (LVisits /h Day). **D** Time course of Licks per visit with licks (Licks/LVisits). Mice were 11-13 months old at the start. The tasks are described at the bottom of the graph and the periods shaded in different colours. Further details about the tasks and IntelliCage abbreviations are shown in Suppl. Tables 2, 3. The time course of IntelliCage data of young mice is shown in Suppl. Figure S7. The data show means ± sem of 8-9 female mice per group. The fluctuation of the behaviour in A reveals nighttime and daytime differences (12h Bins) and show the circadian rhythm. Data were compared with 2-way ANOVA for “time” X “genotype” and posthoc comparison for “genotype” with adjustment of alpha according to Šidák. Throughout all tasks except FA progranulin deficient mice made more LVisits and licks showing the compulsiveness

The biological studies showed that neuronal restoration of progranulin rescued some synapses and axonal / neuronal genes but had no major impact on microglia driven neuroinflammation except the preservation of some homeostatic microglia marker genes in ex vivo primary microglia and partial suppression of a small *Gpnmb*-positive microglia subpopulation that is known for its high phagocytic activity [50, 58]. We next assessed in longitudinal behavioral studies if such subtle differences at the biological level manifested in attenuation of FTD-like behavioral deficits. We had found previously that the onset of behavioral abnormalities in PGRN KO mice is at about 6 months of age. Therefore, we started the first round of IntelliCage observations at 3 months of age and kept the mice in the IntelliCages, until the first symptoms manifested. The time course is shown in Suppl. Fig. S7A-D. The most sensitive parameter is an increase of the frequency of corner visits with licks (LVisits/h) (S7B), and in parallel, a decrease of Nosepokes per Visits showing a reduction of thorough exploration and loss of attention (S7C). The numbers of licks were not yet affected at this age. The mice returned to the IntelliCage at about 1 year of age. Some had to be replaced, and the corresponding time course of key behavioral parameters is shown in Fig. 8. LVisits/h were mostly increased both in PGRN KO and NesGrn KOBG mice, stronger during daytime than at night showing strong overactivity during the “sleep-time”. The numbers of Licks/Visit were strongly increased throughout all tasks in NesGrn KOBG mice. In PGRN KO mice, Licks/Visit were increased only in the adaptation periods but not in learning tasks. The licking exceeds the needs and indicates the compulsive consumption of fluids. Although overall similar in PGRN KO and NesGrn KOBG mice, it appears that the NesGrn KOBG had a stronger disease phenotype than the PGRN KO mice.

### Multi-parameter analysis of IntelliCage behavior shows no advantage of NesGrn KOBG

**Fig. 9:**
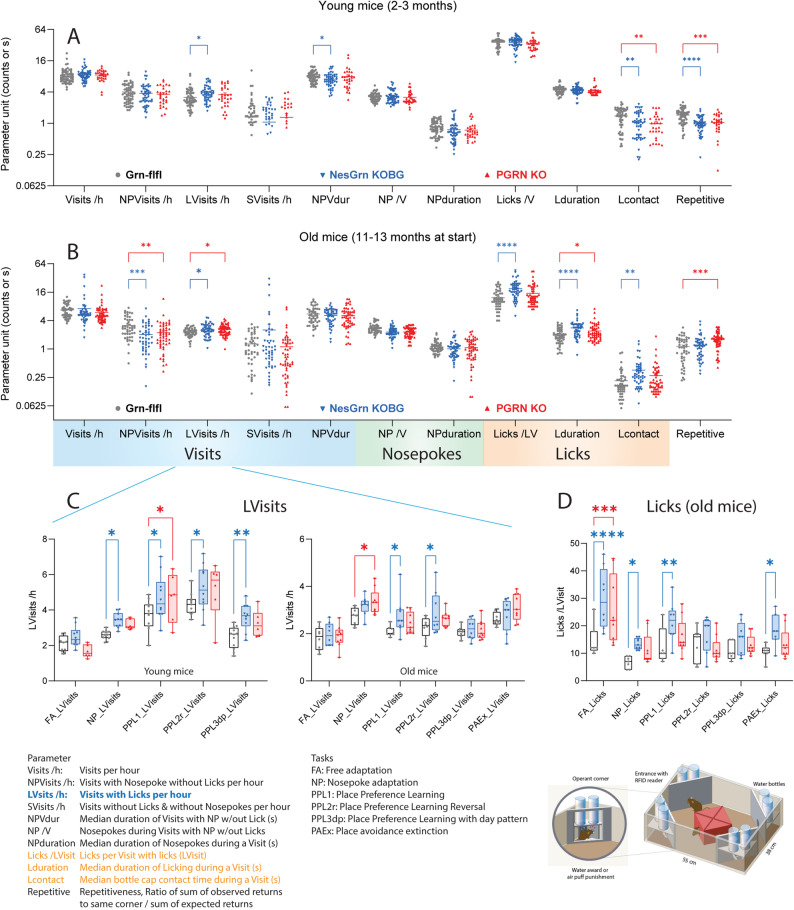
Multiparameter analysis of behavioural features in young and old mice in IntelliCages **A, B**: Multiparameter analysis of different IntelliCage behavioural features for young (**A**) (2-3 months at start) and old mice (**B**) (11-13 months at start). For each mouse, five (young) or six (old) average values were obtained for each of the sequential tasks, which were “Free adaptation” (FA), Nosepoke adaptation (NP), Place preference learning-1 (PPL1), Place preference learning-2 reversal of 1 (PPL2r), Place preference learning-3 with day pattern (PPL3dp), Place avoidance extinction (PAEx, only in old mice). Therefore, each mouse is represented by five (young) or six (old) scatters showing the behaviour of the specific mouse in each task. The line is the average. Data were compared with 2-way ANOVA for the factors “IC-parameter” X“genotype” and posthoc comparison for “genotype” with adjustment of alpha according to Šidák. P*<0.05, **<0.01, ***<0.001, ****<0.0001. **C, D**: The frequency of LVisits (Visits with Licks, **C**) and the Licks/Visit (**D**) differed most between genotypes and are therefore shown for each task. Statistics as in A, B. Licks were particularly high in FA, where all doors were open allowing licking ad libitum. During learning tasks lick duration was restricted by the door closing time of 5 s. Further details about the tasks and IntelliCage abbreviations are shown in Suppl. Tables. The data are from *n ***=**11 Grn-flfl, *n *= 10 NesGrn KOBG, and *n* = 6 PGRN KO mice. All mice were female

To compare multiple behavioral features, the 24h- or 12h-interval behavior was averaged over the duration of the respective task, and the task averages were pooled and presented as scatter plots in Fig. 9A, 9B. As suggested in the time courses, LVisits/h were increased (Fig. 9C) and Nosepokes reduced, and all licking parameters increased in old mice (Fig. 9B). Young progranulin-deficient mice behaved similarly to the controls except for LVisits and repetitive behavior. The latter indicates how often mice return to the same corner within a short time window. It is “smart” if the corner provided reward, if not, it means insistence or inflexibility or fixation to spontaneous corner preferences. In combination with the other parameters and the age of the mice, reduced repetitive behavior in young mice may be interpreted as first sign of overactivity. It was transient and no longer evident in old mice or inverted for old PGRN KO mice. Detailed analysis of LVisits for each task in young and old mice (Fig. 9C) shows the age-dependent decline in all mice and the pathological increase of LVisits in both knockout lines. Pairwise comparisons did not show significant differences between NesGrn KOBG and PGRN KO mice.

**Fig. 10:**
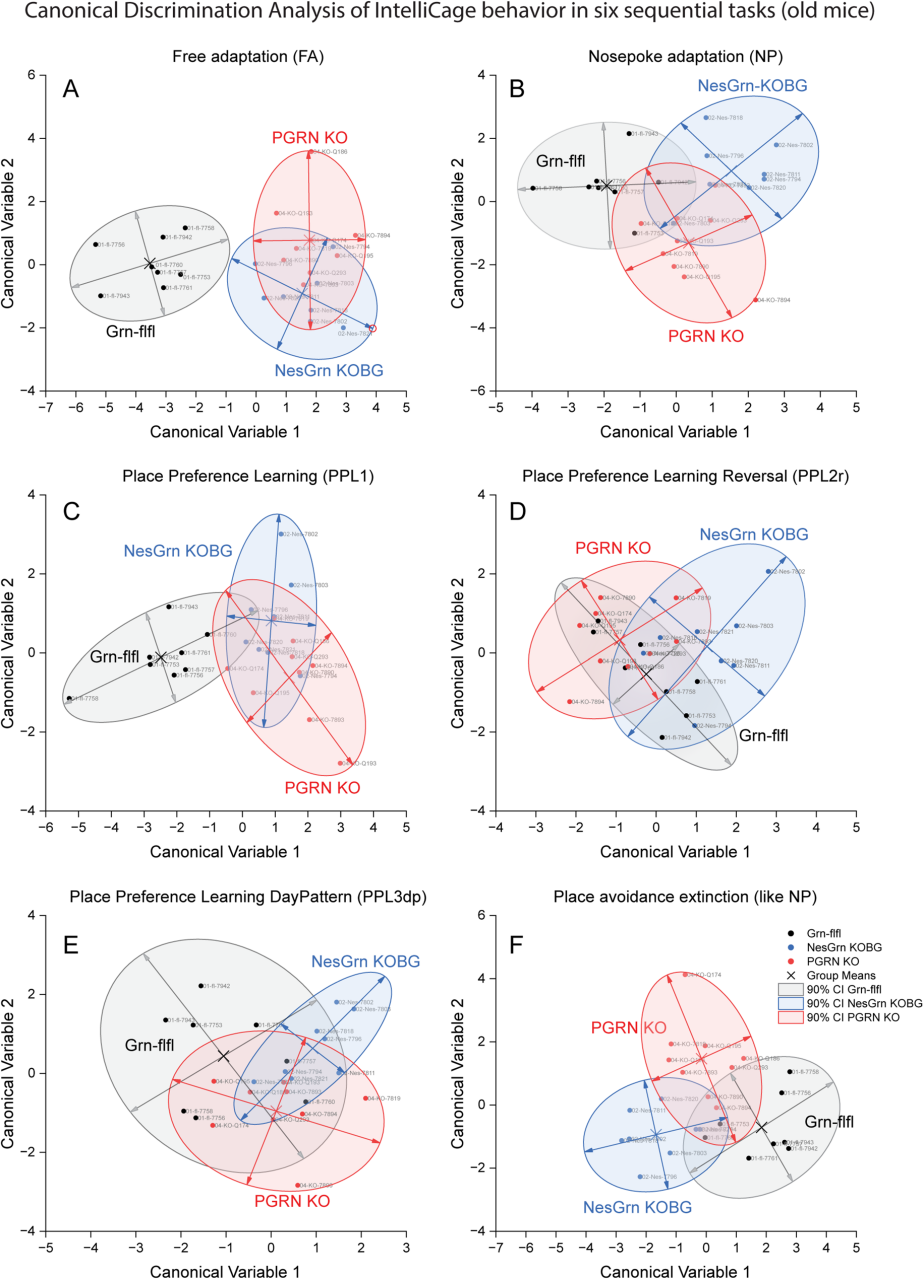
Canonical Discrimination Analysis of IntelliCage behaviour in six sequential tasks (old mice, 11-13 m at start). Behavioural readouts as shown in Figure 6 were submitted to Canonical Discrimination analysis for each task to reduce dimensionality and assess relatedness. The graphs show score plots for CanDisc factor-1 versus factor-2, the scatters are the mice, and the circle is the 90% confidence interval (CI) for prediction of group membership. **A** Free adaptation; **B** nosepoke adaptation, **C** Place preference learning (PPL1); **D** Place preference learning reversal (PPL2r); **E** Place preference learning with day pattern (PPL3dp); **F** Place avoidance extinction (PAEx)

**Fig. 11:**
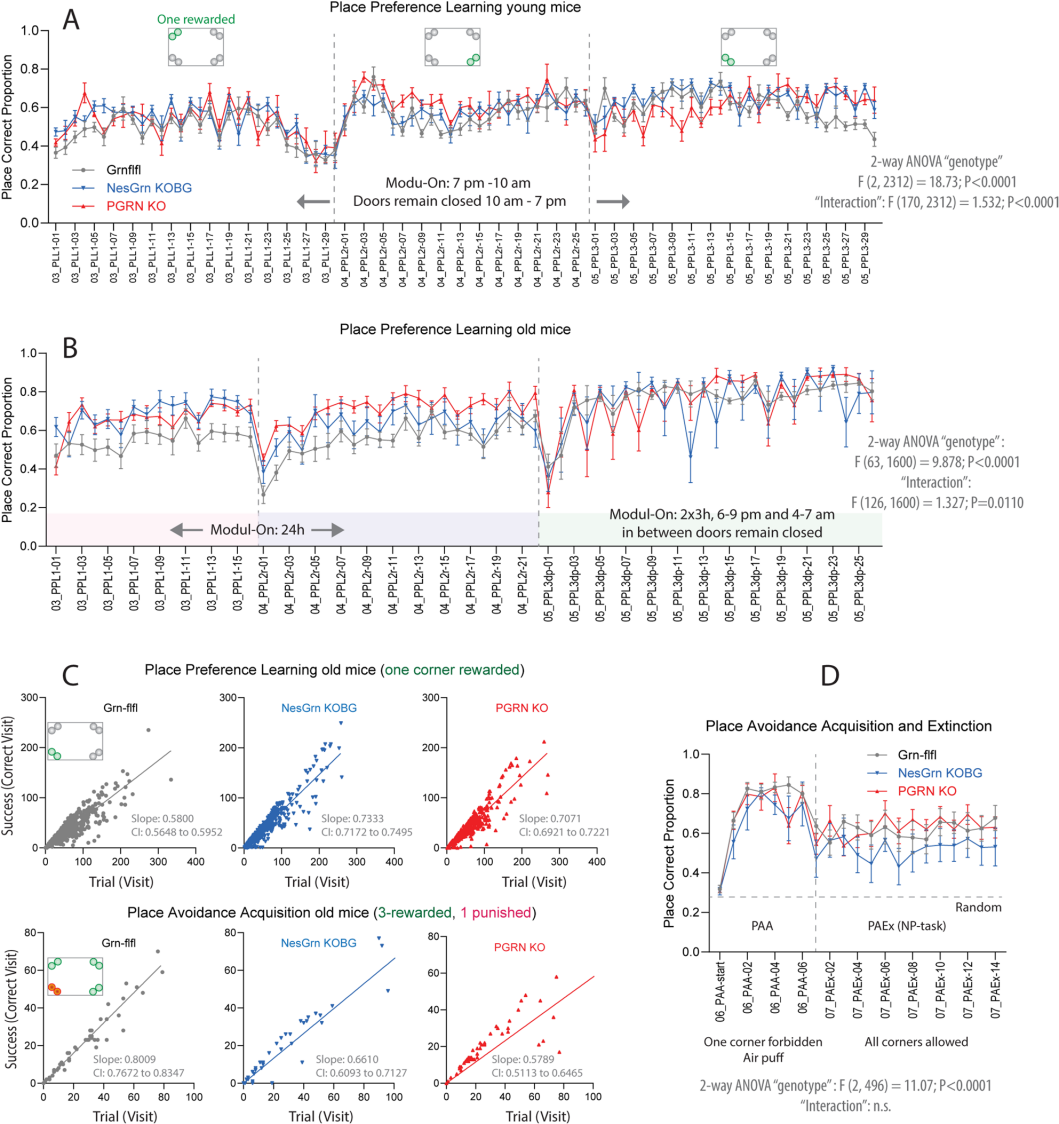
Comparison of accuracy of corner visits as indicator of learning and memory **A**: Time course of correct corner visit proportion relative to all visits during place preference learning tasks in IntelliCages in young mice (2-3 months at start). In young mice, the preference learning tasks were done with a nighttime “Module-On” schedule. During the days, the doors remained closed. **B**: Corresponding time course for old mice (11-13 months at start). In old mice, the modules were active for 24h during PPL1 and PPL2r and followed a 2x3h schedule in PPL3dp. In PPL1 and PPL2r progranulin deficient mice showed a seemingly better performance than the controls reflecting a higher chance of learning owing to the compulsiveness-driven high visiting frequency.**C**: Linear regression analysis of the steepness of the learning curves in place preference learning tasks. The success (correct corner visit) is plotted against the trial (visit). A steep slope reveals fast learning. As seen in B, progranulin deficient mice have a steeper learning curve in reward-based tasks, but they fall behind in the avoidance tasks which is not driven by compulsiveness. **D**: Time course of the proportion of correct corner visits in place avoidance acquisition and extinction tasks. NesGrn KOBG mice lose the unpleasant memory faster than PGRN KO or Grn-flfl mice

To assess if and how the groups could be separated and group membership predicted by behavior, behavioral parameters were submitted to Canonical Discrimination analysis for each task (Fig. 10, Suppl. Fig. S8 for young mice). The score plots show that Grn-flfl can be separated from NesGrn KOBG and PGRN KO but the latter two are mostly overlapping. In young mice, separation of the genotype-clusters is strongest in the difficult PPL reversal tasks (PPL2r, PPL3), whereas the old mice show stronger differences during adaptation and easy place preference learning (PPL1). This paradox is caused by the physiologic overall decrease of corner visits in old mice which reduces the chance of learning. Grn-flfl mice are more affected by this age-dependent decline than progranulin knockout mice who maintain pathologically high activity and corner visits owing to the compulsiveness. Therefore, at old age, both NesGrn KOBG and PGRN KO mice have a learning advantage over Grn-flfl mice in reward-based tasks (Fig. 11), which agrees well with our previous studies using touchscreens where a correct response is rewarded with sweet fat milk [59]. The advantage is lost when the controls are forced to increase their activity in the more difficult tasks.

### Learning and memory

To assess differences in learning and memory behavior, time courses of the accuracy of corner visits (proportion of correct visits) showing reward-based learning and the steepness or learning curves were compared (Fig. 11). Young mice were all similar. Old PGRN KO and NesGrn KOBG mice showed a higher proportion of correct corner visits in the first two preference learning tasks (PPL1, PPL2r) which was lost in the last PPL3dp task which is caused by the additional challenge of the day pattern with restricted Module-active times, which forced the Grn-flfl controls to increase their activity (they caught up) and limited the learning advantage of progranulin knockout mice. The absolute number of correct visits drops in this period (Suppl. Fig. S9). Analysis of the learning curves (Fig. 11C) confirms a steeper (faster) reward-based learning of PGRN KO and NesGrn KOBG mice but in avoidance-based learning, the Grn-flfl controls were superior. NesGrn KOBG mice lost the avoidance memory faster than the other two groups. Overall, learning and memory were equal in PGRN KO and NesGrn KOBG mice.

## Discussion

In this study, we generated mice with Nestin-Cre-driven PGRN restitution in neurons in a full progranulin knockout mouse (NesGrn KOBG) to rescue PGRN in neurons but not in microglia. We show that NesGrn KOBG mice develop a PGRN-loss-associated FTD-like phenotype, which was comparable to the phenotype of full PGRN KO mice, albeit Nestin driven rescue of progranulin in neurons partly attenuated synaptic loss, which is caused by excessive microglia-mediated synaptic pruning [45]. Our transcriptomic and lipidomic studies suggest that partial synaptic preservation was mediated by reduced axonal presentation of phosphatidylserine eat-me signals rather than caused indirectly by neuron-to-microglia sharing of progranulin and thereby silencing of microglia. The lipidomic screen did not differentiate between PS at inner or outer membranes, but exposed PS may result in higher analytical recovery. In combination with the histology results, lower PS in NesGrn KOBG than PGRN KO brain points at an attenuation of pathologic neuronal PS presentation. However, the observed partial neuronal "cure" was not sufficient to prevent astrogliosis and microgliosis and behavioral pathology. 

Supporting this interpretation, bulk transcriptomic, histology and microglia morphology studies all show that microglia as such were not co-cured by neuronal rescue of progranulin. At the molecular level, the neuronal rescue of progranulin prevented a loss of several genes involved in maintenance of axonal structures and dynamics. The result supports the histologic dendritic spine preservation and higher density of synaptic structures in immunofluorescence studies. However, the neuroinflammation with microgliosis and astrogliosis did not differ between PGRN KO and NesGrn KOBG mice, confirming on the one hand that our selective neuronal restoration strategy was successful but on the other hand suggesting that exclusive or predominant neuronal restoration did not provide sufficient or effective progranulin to co-prevent the microglia-mediated perpetuation of the pathology as was hoped for. Encouragingly though, snRNA sequencing studies and rtPCR of isolated primary microglia revealed that neuronal rescue effectively suppressed the emergence of a small *Gpnmb*-positive microglial subpopulation with reportedly high phagocytic activity [46]. These findings suggest that this microglial subset arises in response to synaptic "eat-me" signals and could potentially be prevented through early restoration of neuronal progranulin levels.

Nonetheless, the neuron-only restoration of progranulin did not prevent the FTD-like phenotype although the Nestin-promoter driven Cre ensured an early embryonic onset of neuronal progranulin expression, which was maintained throughout life. The most dominant behavioral FTD-like features are overactivity and compulsive grooming, licking and drinking. Throughout different IntelliCage tasks these key features did not differ between the full PGRN KO and NesGrn KOBG mice. There was also no difference in cognitive performance. A dementia-like cognitive deficit manifested only in aversive learning tasks. In reward-based tasks, compulsiveness creates a high learning drive and therefore mostly outweighs memory deficits. In agreement with our previous studies [37, 59], progranulin deficient mice were therefore more successful than control mice in reward-based place preference learning. PGRN KO and NesGrn KOBG mice shared this phenotype, at least in female mice, which were used in the IntelliCage experiments to avoid mouse fights and dominance behavior which may cause harm to inferior mice. The necessary restriction to female mice for the behavior IC studies is a limitation, but so far, the pathology of progranulin deficiency in mice appears to affect male and female mice equally. In view of the age-dependent progressive nature of the pathology caused by progranulin deficiency we used mostly aged to old mice for our experiments (Suppl. Table 1), which were age-matched between groups as far as possible. Because of the increased frequency of skin lesions in old progranulin deficient mice, a perfect match was not achieved in all experiments. 

Our results partly disagree with a study where progranulin was restored with a neuron-specific adeno-associated virus (AAV) mediated delivery into neurons of the prefrontal cortex in a heterogeneous PGRN mouse [16]. PGRN het mice have no gliosis and about 50% of normal progranulin and are mostly healthy except for subtle social behavioral deficits that manifested in social inferiority behavior in a tunnel test which was attenuated in AAV-*Grn* treated mice [16]. The study used male and female mice but did not analyze differences between sexes [16]. In another study, AAV-*Grn* not only reduced lipofuscinosis in several brain regions of PGRN KO mice but also reduced microgliosis in brain regions distant from the injection site, albeit AAV-expressed progranulin was only detected in neurons, not in microglia. The authors concluded that the observed microglial activation in progranulin deficiency could be improved by targeting neurons only [17]. It is not clear, why AAV-*Grn* was seemingly more effective than the transgenic *Grn*-restoration used in our study. The authors used another knockout line [60] but the constructs and genetic background are very similar to the mice used in the present study [32]. Tissue was obtained 8-10 weeks after AAV-*Grn* injection [17]. It is conceivable that AAV-*Grn *transiently created high local progranulin or a permissive environment supporting uptake by microglia, which might not occur without invasive local brain injection. 

The results of the present study suggest that healthy neurons, or even partly compromised neurons, do not secrete sufficient progranulin to prevent microglia-driven neuroinflammation and microglia-mediated axonal attack that progresses with age in progranulin deficient brain. Alternatively, microglia cannot use the progranulin, which is secreted by neurons either because they do not express the required uptake machinery or receptors, which were found to bind progranulin or because microglia require intracellular progranulin-dependent ER to lysosome signaling, which cannot be substituted by endolysosomal progranulin delivery. Interestingly though, a progranulin-transferrin-receptor-binding fusion construct was able to prevent neuroinflammation [21] even after peripheral delivery to the liver with a liver targeting AAV [20] suggesting that binding to the transferrin receptor permitted BBB transfer and cellular uptake including uptake by microglia, which appeared to be as efficient as brain-injected AAV-*Grn.* The peripheral approach however, might be associated with an increased risk of cancer because progranulin promotes tumorigenesis and cancer progression [61–64]

In line with the AAV-*Grn* replacement studies, our previous studies in a controlled cortical impact model of traumatic brain injury (TBI) showed that even little neuronal progranulin restitution was able to rescue the excessive structural damage and microglial activation that occurs after experimental TBI in full PGRN knockout mice [43], suggesting that injured or dying neurons release sufficient progranulin to prevent the massive brain infiltration by CD68^+^ microglia in the context of TBI, although microglia themselves lacked PGRN. It is also conceivable that microglia receive a progranulin-signal from the cargo of apoptotic bodies [65], which are abundant after TBI. The results of the TBI study suggested that endogenous PGRN expression in microglia was not essential for attenuating structural brain damage after TBI [43]. In contrast, the results of the present study show that although neuronal progranulin restoration was able to preserve several axonal genes, synapses and synaptic spines which were lost in PGRN KO, it did not prevent neuroinflammation, nor did it prevent the behavioral compulsive overactive phenotype which is characteristic for PGRN KO mice. The results suggest that compulsiveness may be driven at least in part by neuroinflammation, which would agree with studies showing that optogenetic activation of microglial subpopulations causes compulsive grooming [66]. We did not observe social inferiority behavior in IntelliCage experiments such as hierarchical corner visits, but the experiments were not designed to provoke stressful social competition, which can be achieved for example by assigning all mice to one rewarding corner and restricting drinking times. In our experiments, four mice each were assigned to one corner, and the overall visit frequency was similar in all groups.

Our findings may have potential therapeutic implications and add to the understanding of cell type-specific functions of PGRN. For slowly progressive neurodegeneration with protracted neuronal death and partial loss of dendrites and synapses it is conceivable that a lasting therapeutic benefit cannot be achieved by restoration of progranulin in neurons only, because it may not resolve the microglial pathology, according to our studies. It may be necessary to combine *Grn*-gene substitution with microglia depletion and replacement for example through genome-edited or allogeneic stem cell transplant, an approach that provided promising results in a progranulin-knockout mouse model [27, 67], and in inherited human leukodystrophy cases [31, 68]. Alternatively, brain penetrant progranulin fusion constructs which facilitate cellular uptake independent of cell type [20, 21], or intranasal nose-to-brain delivery [69] through e.g. engineered progranulin-enriched exosomes might be options to substitute progranulin in neurons and microglia. 

## Supplementary Information

Supplementary Material 1.

Supplementary Material 2.

Supplementary Material 3.

Supplementary Material 4.

## Data Availability

All data that were analysed for the study are presented within the manuscript or supplementary files. The RNAseq data have been uploaded to the GEO repository and are available under the accession number GSE273083. Lipidomic studies of mouse brain are available at BioStudies under the accession number S-BSST2143.
