# Supplementary material for: Selective neuronal restoration of progranulin does not prevent the frontotemporal dementia like-phenotype of progranulin knockout mice
Source: J Neuroinflammation. 2026 Jan 10;23:34. doi: 10.1186/s12974-025-03665-3 (PMC12836895; doi:10.1186/s12974-025-03665-3)
Supplement: Supplementary file 2 — Supplementary Material 2. [file 12974_2025_3665_MOESM2_ESM.pdf]

Supplementary Tables

Suppl. Table S1

Overview of mouse groups, ages and sex in behavioural and biological experiments

Experiment	Readout	Interpretation	Grn-flfl	NesGrn KOBG	PGRN KO
**IntelliCage Young mice**	Activity, Learning and memory	Activity, exploratory behaviour, Learning/memory, social interaction	Female n = 12, age 2-3 months at start	Female n = 10, age 2-3 months at start	Female n = 6, age 2-3 months at start
**IntelliCage Old mice**	Activity Learning and memory	Activity, exploratory behaviour, Learning/memory, social interaction	Female n = 8, age 11-13 months at start	Female n = 8, age 11-13 months at start	Female n = 9, age 11-13 months at start
**snRNAseq**	Single nucleus mRNA	Differential gene expression	Male n = 2, age 23 months	Female n = 3, age 12-18 months	Male n = 3, age 21-24 months
**RNAseq**	mRNA brain	Differential gene expression	Female n = 8, end of IC age 13-15 months	Female n = 7, end of IC age 13-15 months	Female n = 8, end of IC age 13-15 months
**Lipidomic**	Brain lipids	Structural and metabolic brain alterations, lipid-loaded microglia	Male n = 9, age 6 months; Female n = 6, end of IC age 13-15 months	Female n = 7, End of IC age 13-15 months	Male n = 8, age 4-6 months; Female n = 8, end of IC age 13-15 months
**Microglia morphology**	Morphology	Differential cell morphology	Male n = 3, age 14-22 months	Male n = 4, age 18 months	PGRN het male n = 4, age 16 months; PGRN KO f,m n = 3 age 15-16 months
**Gliosis and synapse immuno-fluorescence histology**	Gliosis, microglia and astrocyte marker	Differential cell and synapse area coverage in different brain regions	Female n = 5, age 9-18 months	Female n = 4, age 14-22 months	Female n = 3, age 10-13 months
**rtPCR candidate genes**	Relative gene expression log2(Fold change) vs. control	Differential gene expression of isolated microglial cells	Male n = 4, age 3-10 months	Male n = 6, age 3-10 months	PGRN KO female n = 5, 10-17 months; male n = 5, 6-13 months<br>PGRN het male n = 3, 4-5 months
**Spine density**	Spine density	Differential spine density	Female n = 5, age 9-18 months	Female n = 4, age 14-22 months	Female n = 3, age 10-13 months

Suppl. Table S2

IntelliCage Tasks (old mice*)

Experiment	Duration	Task description	Readouts, interpretation
**Free adaptation (FA)**	7 days	General habituation to the cage with open access to every corner, all doors open, and water/food ad libitum	Exploratory behaviour, activity, circadian rhythms, social interaction
**Nosepoke adaptation (NP)**	8 days	The first nosepoke of a visit opened the door for 5 s. To drink more, the mouse has to start a new visit.	Exploratory behaviour, activity, circadian rhythms, social interaction
**Place preference learning (PPL1)**	9 days	Mice were allowed to drink in one of the 4 corners. The first correct nosepoke of a visit opened the door for 5 s. To drink more, the mouse has to start a new visit. 4 mice were assigned to one corner. Learning was supported by LED	Spatial preference learning by reward, exploratory behaviour, activity, social interaction
**Place preference reversal 1 (PPL2r)**	13 days	Equal protocol as in “place preference learning” but with the opposite corner as the correct corner. Learning was supported by LED	Cognitive flexibility of Reversal Learning requires the dorsal and ventral hippocampus and their functional interactions with the prefrontal cortex (Vila-Ballo, Mas-Herrero et al. 2017, Avigan, Cammack et al. 2020)
**Place preference reversal 3 (PPL3dp)**	14 days	Equal protocol as in “place preference learning” but with a new correct corner and day pattern where the learning modul was only active 2x3h per day. Outside the doors remained closed. Learning was supported by LED	Reversal learning with restricted “Modul-active-times”
**Place avoidance acquisition (PAA)**	3 days	Mice had to avoid one corner. A NP in this corner was punished with an air-puff and a red LED. 4 mice were assigned to one forbidden corner.	Spatial avoidance learning by punishment is sensitive to genetic differences and hippocampal lesions (Voikar, Colacicco et al. 2010)
**Place avoidance extinction (PAAex)**	8 days	NP would open the doors in each corner without punishment. Only the red LED still indicated the previously forbidden corner.	Retention of avoidance behaviour. Avoidance memory. Duration of the avoidance behaviour reflects caution over curiosity

*The protocols in young mice were similar with some differences for the task duration and PAA/PAEx was only done in old mice.

Suppl. Table S3

Abbreviations of behavioural parameters of IntelliCage experiments

Parameter	Description
**Visits**	Visits / h
**NPvisits**	Visits with Nosepoke without Licks / h
**Lvisits**	Visits with Licks / h
**SVisits**	Visits without Licks and without Nosepokes / h
**NPVdur**	Median duration of Visits with NP w/out Lick (s)
**Nosepokes (NP)**	Mean number of Nosepokes during Visits with NP w/out Licks
**NPduration**	Median duration of such Nosepokes during a Visit (s)
**Licks**	Median number of Licks per Visit
**Lduration**	Median duration of Licking during a Visit (s)
**Lcontact**	Median bottle cap contact time during a Visit (s)
**Nocturnal**	Log(Visit frequency during dark phase / Visit frequency during light phase)
**Repetitive**	Repetitiveness, log(sum of observed returns to same corner / sum of expected return)
**IVI**	Intervisit intervals (s) i.e. time from end of visit to start of next corner visit
**IVIrepdens**	Intervisit intervals (s) for repeated use of the same corner
**Unevenness**	Describes the relative use of corners, ranges from 0-1 (0=equal use of 4 corners, 1=exclusive use of 1 corner)
**Sidedness**	Ratio of visits with first left versus first right NP of visits with NPs
**Mesor**	**M**idline **e**stimating **s**tatistic **o**f **r**hythm. The mesor is a circadian rhythm-adjusted mean based on the parameters of a cosine function fitted to the raw data of the visits.
**Amplitude**	Difference between Mesor and Peak activity
**Acrophase**	Time to maximum activity after Light Off (Light off set to 0)

Suppl. Table S4

Used primer in rtPCR candidate genes experiments

Gene name, (amplicon size, annealing temperature)	Oligonucleotide sequences 5'–3'<br><br>(fw: forward, rev: reverse)	Gene bank number
***Ppia* (144 bp, 60°C)**	fw- GCTGGACCAAACACAAAACGG<br>rev- GCCATTCCTGGACCCAAAAC	NM_008907
***Grn* (171 bp, 60°C)**	fw- CTGCCCGTTCTCTAAGGGTG<br>rev- ATCCCCACGAACCATCAACC	NM_008175
***Gapdh* (100 bp, 60°C)**	fw- CCTCGTCCCGTAGACAAAATG<br>rev- TCTCCACTTTGCCACTGCAA	NM_001289726
***Nos2* (127 bp, 60°C)**	fw- GTTCTCAGCCCAACAATACAAGA<br>rev- GTGGACGGGTCGATGTCAC	NM_010927
***Tgfb1* (133 bp, 60°C)**	fw- CTCCCGTGGCTTCTAGTGC<br>rev- GCCTTAGTTTGGACAGGATCTG	NM_011577
***P2ry12* (77 bp, 60°C)**	fw- QuantiTect Panel Qiagen<br>rev- QuantiTect Panel Qiagen	NM_027571
***Ube2d2* (125 bp, 60°C)**	fw- TGTCCATCTGTTCTCTGTTGTGTG<br>rev- ATACTTCTGAGTCCATTCCCGC	NM_019912
***Csf1r* (96 bp, 60°C)**	fw- QuantiTect Panel Qiagen<br>rev- QuantiTect Panel Qiagen	NM_001037859
***Cx3cr1* (63 bp, 60°C)**	fw- QuantiTect Panel Qiagen<br>rev- QuantiTect Panel Qiagen	NM_009987
***P2rx4* (73 bp, 60°C)**	fw- QuantiTect Panel Qiagen<br>rev- QuantiTect Panel Qiagen	NM_011026
***Trem2* (132 bp, 60°C)**	fw- QuantiTect Panel Qiagen<br>rev- QuantiTect Panel Qiagen	NM_031254
***Apoe* (135 bp, 60°C)**	fw- QuantiTect Panel Qiagen	NM_009696

	rev- QuantiTect Panel Qiagen	
***Gpnmb* (134 bp, 60°C)**	Fw- TCTATCCCTGGCAAAGACCCAG<br>Rev- ATTGGCTTGTACGCCTTGTG	NM_053110

Suppl. Table S5

Used antibodies in microglia morphology and gliosis histology experiments

**Primary antibodies**				
**Primary antibody**	Host	IHC, dilution	Manufacturer	RRID
**Iba1**	Rabbit	1:400	FUJIFILM Wako Chemicals Europe GmbH, Neuss, Germany	AB_839504
**GFAP**	Rat	1:200	Invitrogen by ThermoFisher Scientific, Waltham, USA	AB_2532994
**CD68**	Mouse	1:200	Bio-Rad Laboratories, Hercules, USA	AB_2291300
**CD11b**	Rat	1:200	Bio-Rad Laboratories, Hercules, USA	AB_321292
**PSD95**	Rabbit	1:500	Synaptic Systems GmbH, Göttingen, Germany	AB_2832231
**SV2**	Mouse	1:50	Developmental Studies Hybridoma Bank, Iowa City, USA	AB_2315387
**Secondary antibodies**				
**Fluorophore**	anti-	IHC, dilution	Manufacturer	RRID
**Alexa 488**	Rat	1:500	Invitrogen/Life Technologies	AB_2535794
**Alexa 647**	Rabbit	1:500		AB_2535813
**Alexa 488**	Mouse	1:1000		AB_2534069
