# Supplementary material for: Selective neuronal restoration of progranulin does not prevent the frontotemporal dementia like-phenotype of progranulin knockout mice
Source: J Neuroinflammation. 2026 Jan 10;23:34. doi: 10.1186/s12974-025-03665-3 (PMC12836895; doi:10.1186/s12974-025-03665-3)
Supplement: Supplementary file 3 — Supplementary Material 3. [file 12974_2025_3665_MOESM3_ESM.pdf]

Supplementary figures and legends to

Selective neuronal restoration of progranulin does not prevent the frontotemporal dementia like-phenotype of progranulin knockout mice

Marc-Philipp Weyer[1], Lisa Hahnefeld[1,2], Luisa Franck[1], Matthias Klein[3], Gerd Geisslinger[1,2], Michael K.E. Schäfer[4,5], Irmgard Tegeder[1]

Suppl. Figure S1

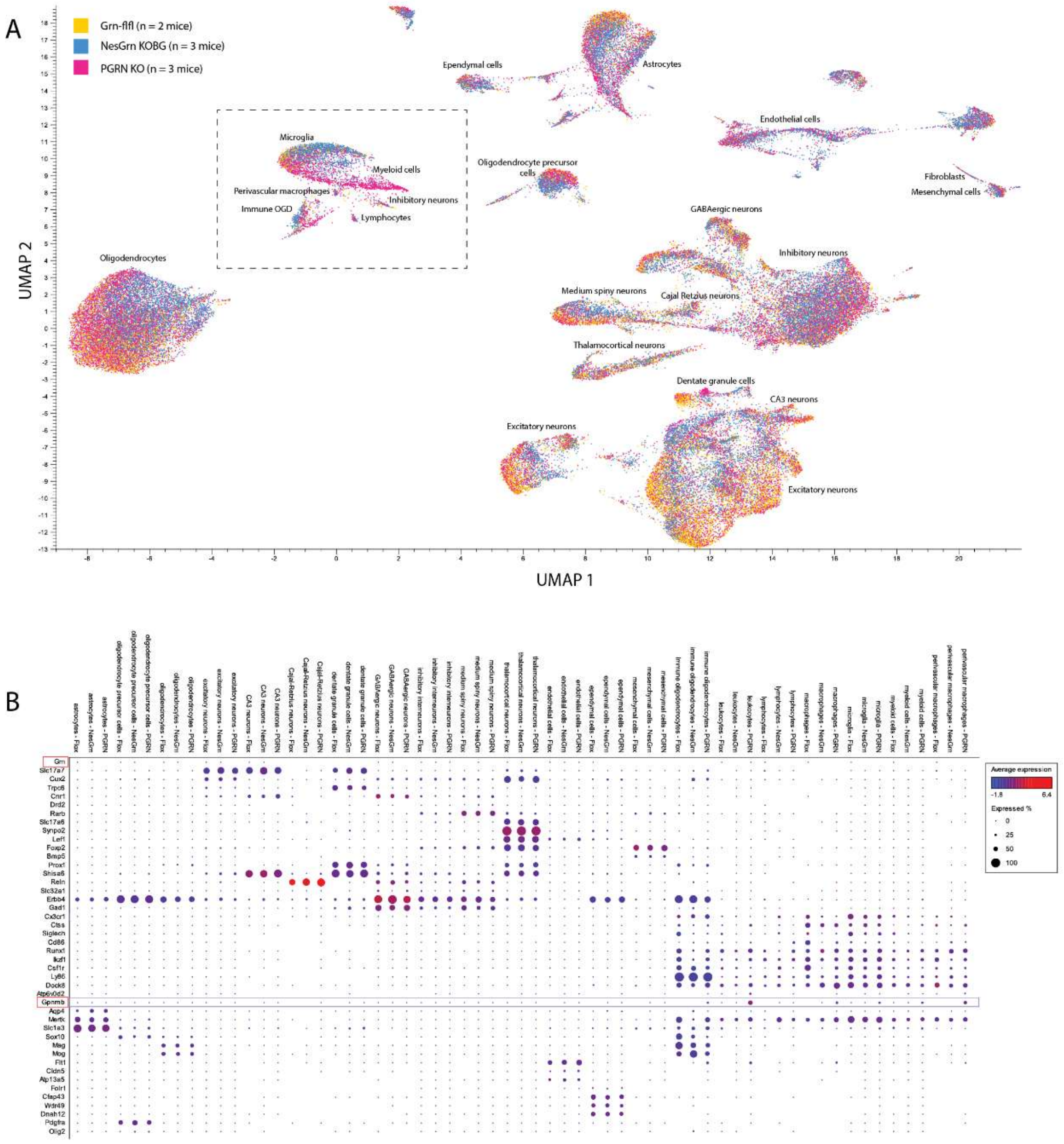

Suppl. Figure S1

Single nucleus mRNA sequencing (sn-RNAseq) analysis of mouse brain in old Grn-flfl, NesGrn KOBG and PGRN KO mice.

A: UMAP overview of cell clusters. Cells were annotated based on the expression of marker genes and known cell-type signatures using the cell type prediction tool implemented in the CLC Genomics workbench single cell analysis plugin. The dashed rectangle indicates the microglial-myeloid population, which was submitted to further analysis presented in the main body in Figure 2.

B: Dot plot of marker gene expression of the cell clusters presented in A. Gpnmb is highlighted representing a microglial/myeloid cell subpopulation, which occurred only in PGRN KO mice. These cells were Gpnmb and Atp6v0d2 and mostly Lgals3 positive.

Suppl. Figure S2

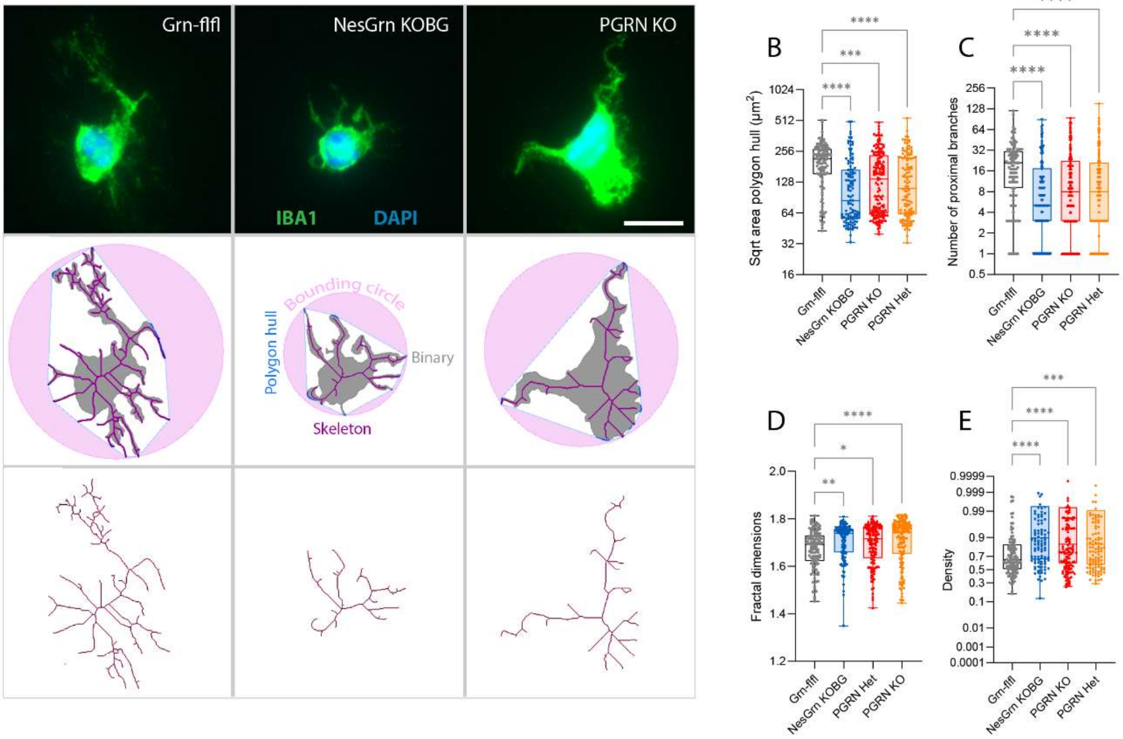

Suppl. Figure S2

Microglia morphology and feature quantification

A: The upper row shows exemplary images of primary microglia from old mouse brains, stained with anti-IBA1 and DAPI. The bottom row shows the skeleton, and the middle row is an overlay of the binary images, the skeleton, the polygonal hull and the bounding circle which were used for quantification of the morphologic features including size and arborization, branch length and density, and fractal dimensions.

B-E: Quantitative analysis of the microglia size represented by the square root of the polygon area (A), numbers of proximal branches (B), fractal dimension (C) and the density, which is the relative coverage of the bounding circle with cell body and branches. If the microglia is plump with short branches the bounding circle is almost filled completely, i.e. density is high in activated microglia. Fractal dimensions provide an index of microglial branching complexity and number of primary processes [1]. The box-counting method was used to calculate the fractal dimensions.

Each scatter is one microglia cell. The box is the interquartile range, the line is the median, and whiskers show minimum to maximum. A total of 117-148 cells of 3-4 animals per genotype were analyzed.

Data were submitted to Brown-Forsythe and Welch ANOVA and subsequently, Dunnett's T3 multiple comparison test. P*<0.05, **<0.01, ***P<0.001, ****<0.0001.

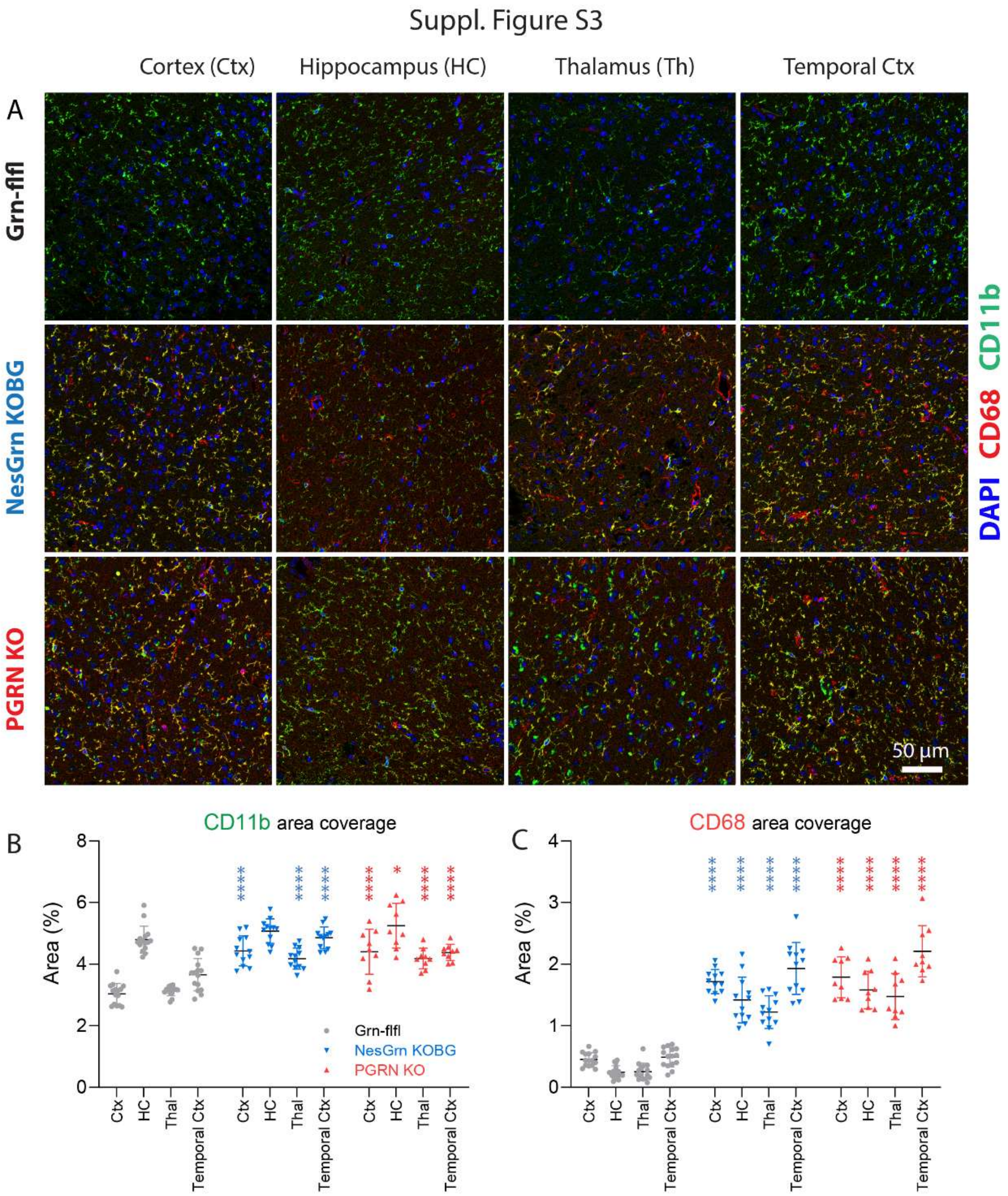

Suppl. Figure S3

Immunofluorescence analysis of microgliosis in cortex, hippocampus and thalamus (CD11b, CD68)

A: Immunofluorescent images of CD11b and CD68 immunoreactive microglia in the cortex, hippocampus, thalamus and temporal cortex of Grn-flfl control mice, of NesGrn KOBG mice and of full PGRN KO mice. DAPI is used as nuclear counterstain. A more detailed analysis of n = 3-5 mice is shown in Suppl. Histology file.

B, C: For quantitative analysis, images were converted to binary images using auto-threshold in FIJI ImageJ, and the relative area covered by specific immunofluorescence for either CD11b or CD68 was used for statistical comparison. The scatters show results per image of n = 3-5 mice per genotype. The line is the average, and whiskers show the standard deviation. Data were submitted to 2-way ANOVA for "brain region" by "genotype". Each region was then compared to the respective region of the Grn-flfl mice using an adjustment of alpha according to Dunnett. Asterisks reveal significant differences versus Grn-flfl. P*<0.05, ****<0.0001.

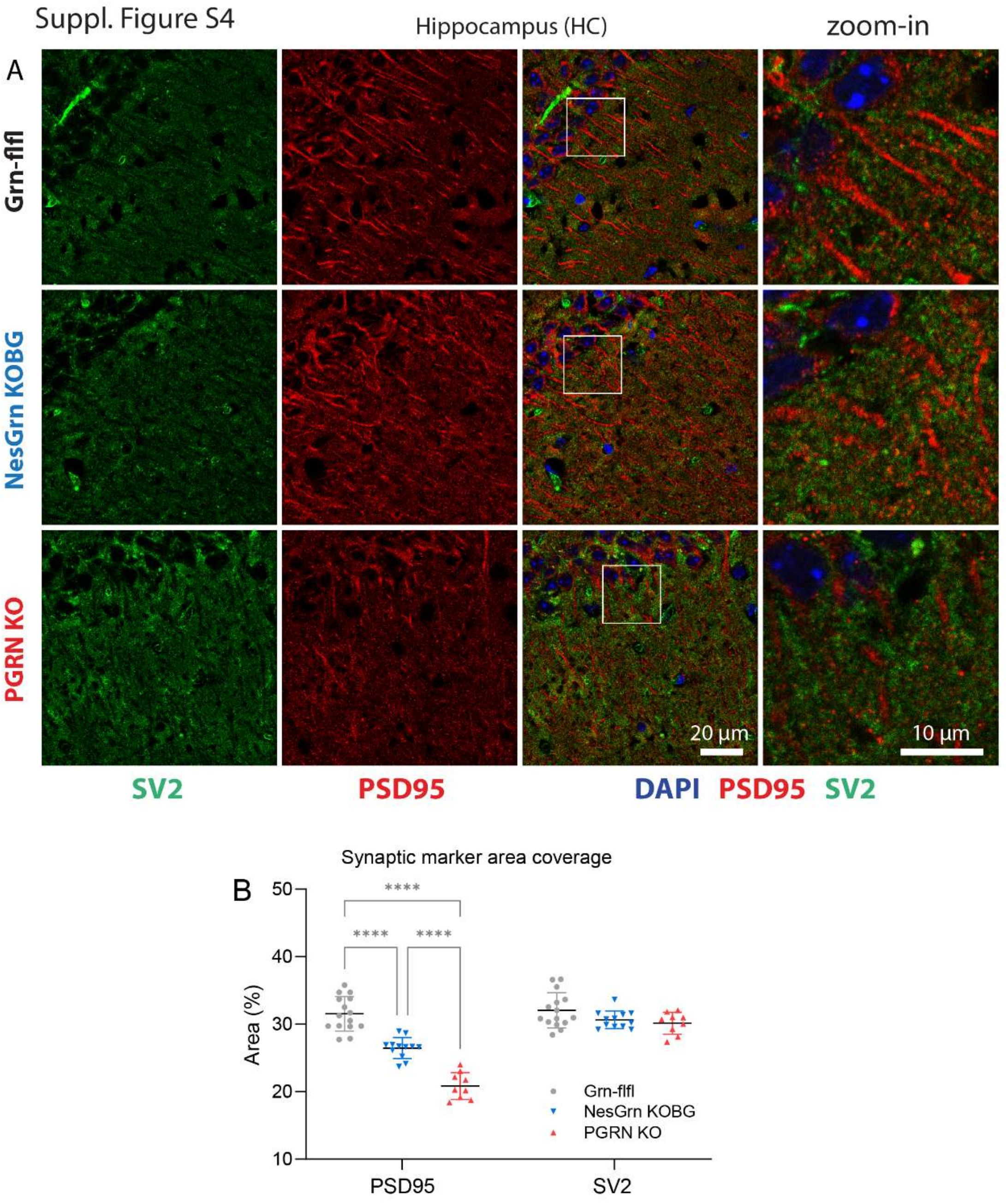

Suppl. Figure S4

Immunofluorescent analysis of synapses in the hippocampus (PSD95, SV2)

A: Immunofluorescent images of the hippocampus from old Grn-flfl, NesGrn KOBG and PGRN KO mice showing Postsynaptic Density Protein 95 (PSD95) immunoreactive postsynaptic structures and Synaptic Vesicle glycoprotein 2 (SV2) immunoreactive presynapses and DAPI as nuclear counterstain. The right panel shows zoom-in images of the rectangles showing in the third column.

B: For quantitative analysis, images were converted to binary images using auto-threshold in FIJI ImageJ, and the relative area covered by specific immunofluorescence for either PSD95 or SV2 was used for statistical comparison. The scatters show results per image of n = 3-5 mice per genotype. The line is the average, and whiskers show the standard deviation. Data were submitted to 2-way ANOVA for “brain region” by “genotype” and subsequent posthoc analysis using an adjustment of alpha according to Tukey. Asterisks reveal significant differences. P****<0.0001.

Suppl. Figure S5

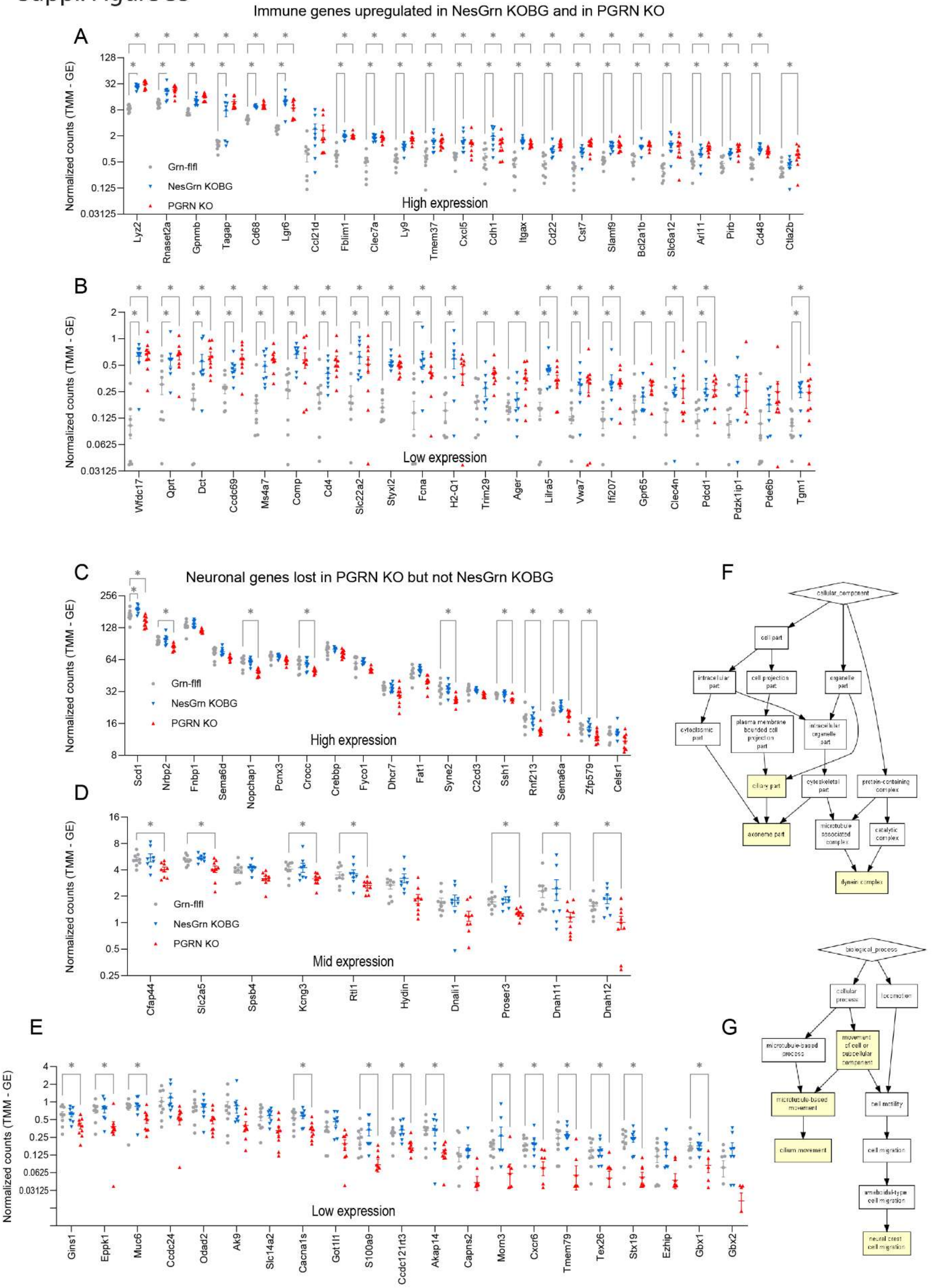

Suppl. Figure S5

mRNAseq candidate differential gene expression in cortical brain of old mice

A, B: Scatter plots show TMM normalized counts of top differentially expressed immune associated genes with high abundance (A) and low abundance (B). Genes were sorted (large to small) on a differential expression score obtained by -Log10 of ANOVA P-value x Abs (Log2(Fold change)), and the top candidates were selected, and sorted according to abundance. The scatters are mice, the line is the mean, and the whiskers show the standard deviation. The asterisks indicate FDR adjusted discoveries.

C, D, E: In analogy to A, B the scatter plots show TMM normalized counts of genes which are downregulated / lost in PGRN KO mice but not regulated in NesGrn KOBG mice. These are mostly neuronal genes.

F, G: Genes lost in PGRN KO but normal/restored in NesGrn KOBG were submitted to Gene Ontology enrichment analysis for cellular component (CC) and biological process (BP) using the GORILLA web based tool (https://cbl-gorilla.cs.technion.ac.il/). The analysis shows enrichment of axonal genes, dynamin complex and neural migration.

Further GO analyses are shown below (Suppl. Fig. S5 Extended GO analyses), for which ShinyGO 0.85.1 (https://bioinformatics.sdstate.edu/go/) was used.

Suppl. Fig. S5 Extended GO analyses

Genes upregulated both in PGRN KO and NesGrn KOBG

Top 30

Lyz2	Wfdc17
Mpeg1	Ctsd
Gpnmb	Cst7
Cd68	Cd84
C1qb	C3ar1
Ctss	Hexa
C1qc	Ms4a7
C4b	Serpina3n
Tagap	Csf3r
Tyrobp	Icam1
Gfap	Lgals3
Trem2	Atp6v0d2
Clec7a	Stab1
Ly9	Aif1
Lag3	Cd86

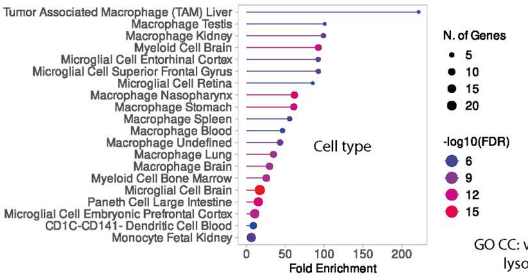

GO CC: vesicle, secretory vesicle, lysosome, granule, ECM

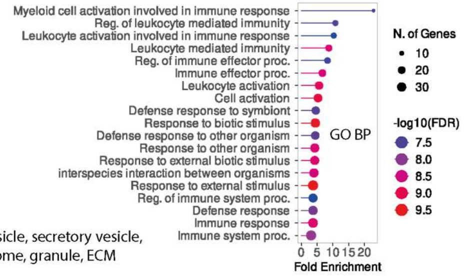

Genes upregulated only in PGRN KO

Top 16

1810062G17Rik
Ptx3
Egr2
Draxin
Cdkn1a
Asb11
Arsi
Thbs4
Fst
Sult1a1
Mterf1b
Pxmp2
Itgb3bp
Pnpla2
Paox
Gadd45b

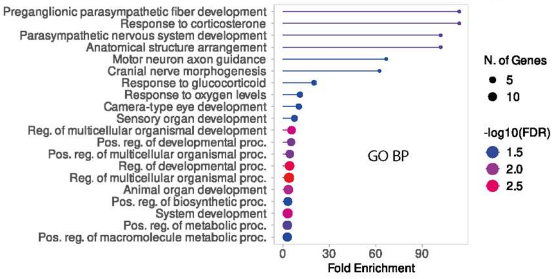

Genes upregulated only in NesGrn KOBG

Top 30

Aqp4	Trf
Hopx	Vwa1
Mmp14	Mrc1
Tgm2	Dab2
Ednrb	Lpcat2
Eng	Myoc
Rbp1	Ogn
Tst	Steap3
Pla2g4a	Vcam1
Efhd1	Ephx2
Ly6a	Thbd
Adamtsl4	P2ry13
Lrp4	Clic4
Cfap54	Kdr
Lrg1	Mrc2

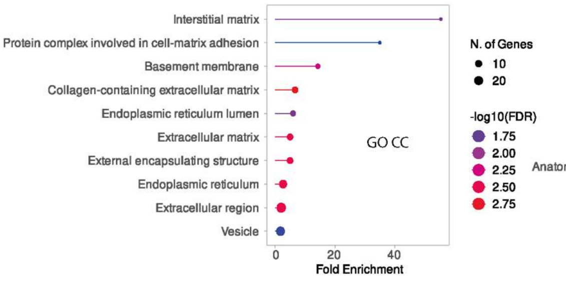

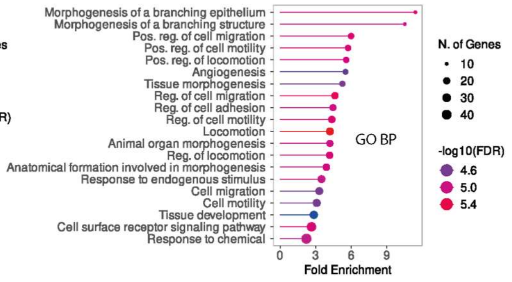

Genes lost only in PGRN (fully or partially restored in NesGrn KOBG)

Top 30

Gbx2	Got1l1
Tex12	Ccdc24
Spata25	Ak9
Oxt	Cfap73
Grn	Htr2b
Tmem79	Pcsk9
Stx19	Cfap141
S100a9	Dnah12
Gbx1	Abcb1b
Capns2	Cacna1s
Lrrd1	Muc6
Wdr49	Dnah10
Lrtm1	Cfap46
Akap14	Cfap44
Tmem174	Dnah7c

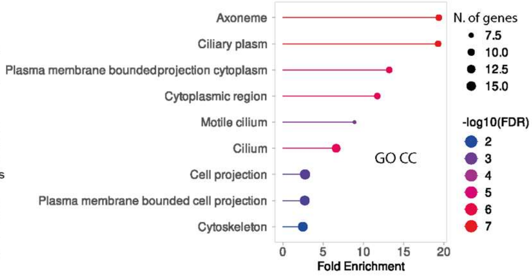

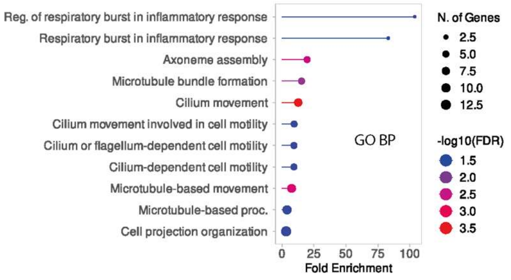

Genes lost only in NesGrn KOBG

Pcp2 (purkinje cell protein 2)

Thumpd

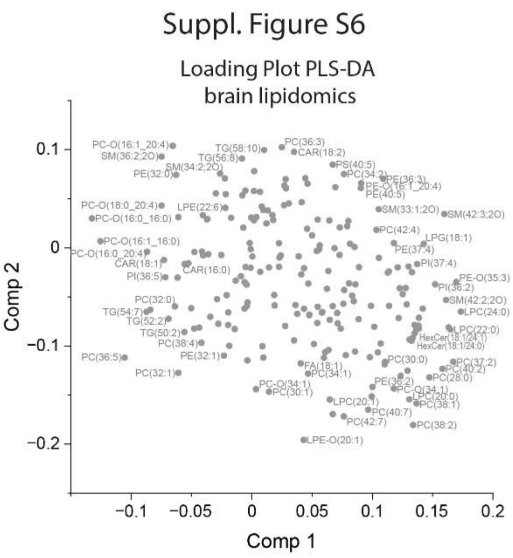

Suppl. Figure S6

Loading plot of Partial Least Square Discrimination analysis of brain lipidomic studies

The scatters show the XY loading coordinates of the PLS-DA component-1 (X) and component-2. The lipids which contributed most to the variability between genotypes are at the outer boundaries of the scatter cloud and are labelled with the lipid species.

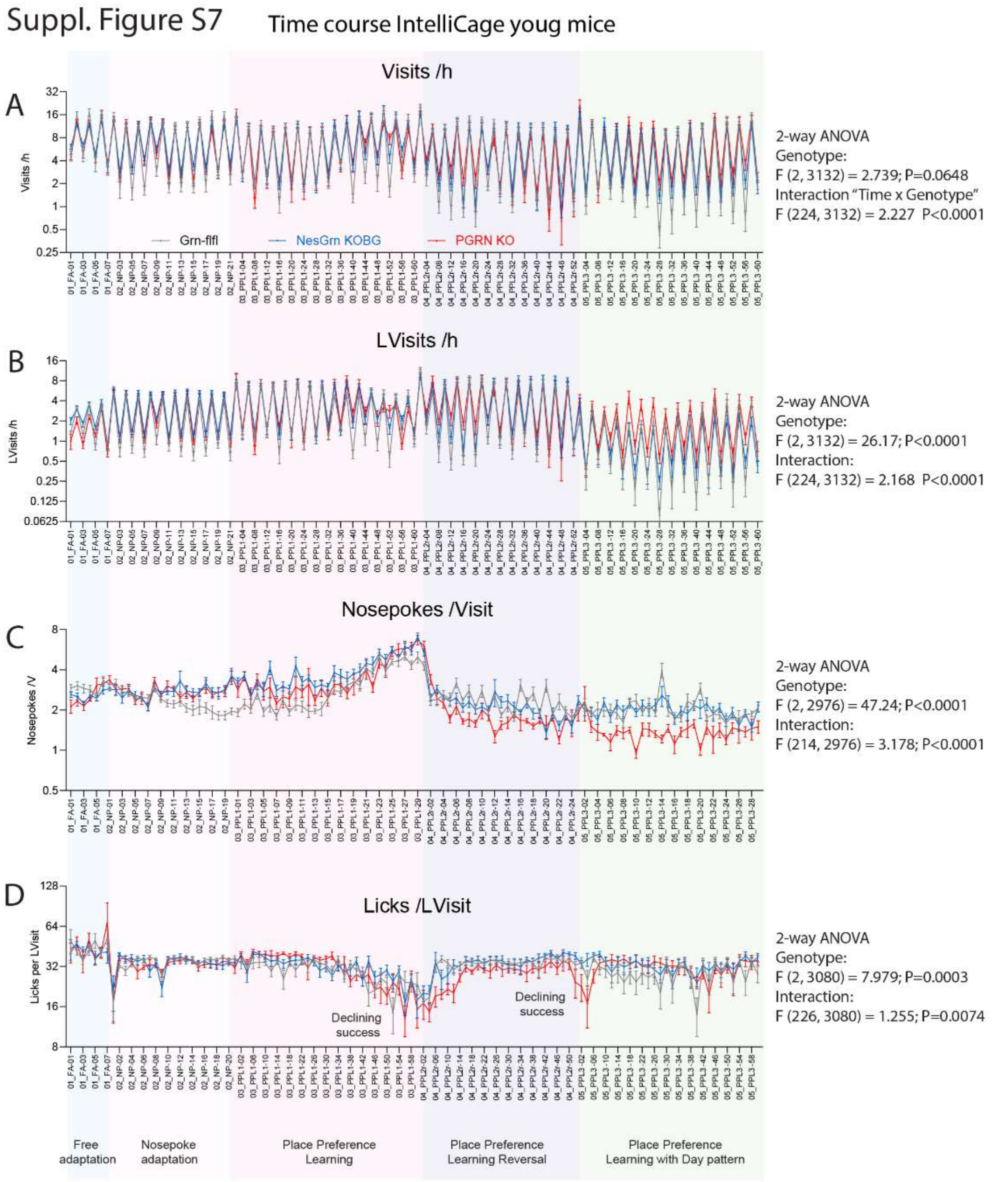

Suppl. Figure S7

Longitudinal behaviour of young Grn-flfl, PGRN KO and NesGrn KOBG in IntelliCages

A: Time course of corner visits per hour (Visits /h) during different tasks in IntelliCages**.**

B: Time course of corner visits with licks per hour (LVisits /h)**.**

C: Time course of Nosepokes per Visit (NP /Visit). The ratio is an indicator of the exploratory activity.

D: Time course of Licks per visit with licks (Licks /LVisits)**.**

The tasks are described at the bottom of the graph and the periods shaded in different colours. Further details about the tasks and IntelliCage abbreviations are shown in Suppl. Tables 2, 3. The data show means ± sem of 12, 10 and 6 female Grn-flfl, NesGrn KOBG and PGRN KO mice, respectively. The fluctuation of the behaviour in A, B reveals nighttime and daytime differences (12h Bins) and show the circadian rhythm. Data were compared with 2-way ANOVA for "time" X "genotype" and posthoc comparison for "genotype" with adjustment of alpha according to Šidák. In the final PPL3 task, PGRN KO mice started to show reduced exploratory activity (NP/Visit, C) but increasing LVisits/h (B) while Licks per se were still normal (D). The change suggests the onset of compulsive behaviour. NesGrn KOBG mice were still normal during the experiment.

Suppl. Figure S8

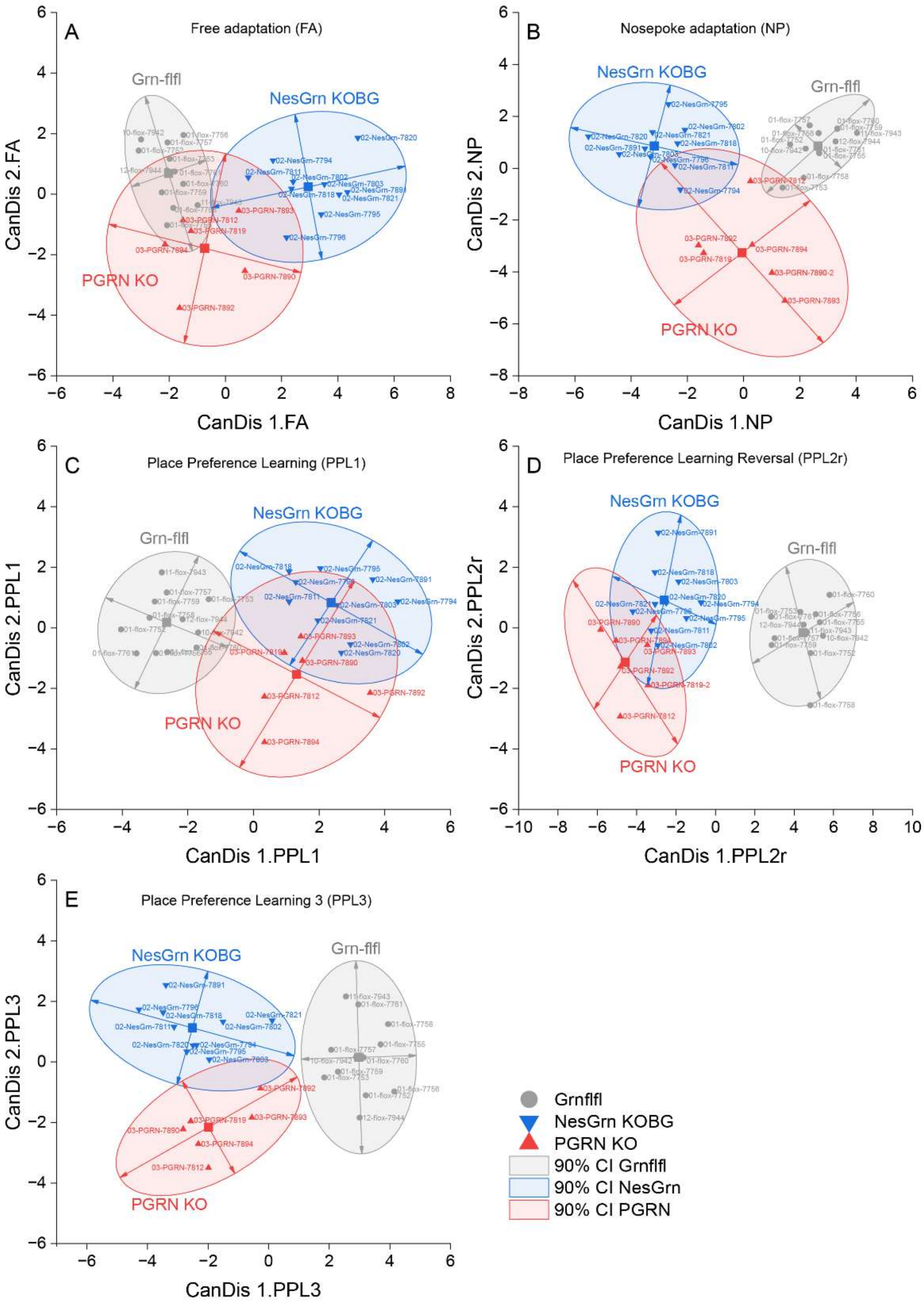

Suppl. Figure S8

Canonical Discrimination Analysis of IntelliCage behavior in five sequential tasks (young mice)

Behavioral parameters are shown in the main body in Figure 6. They were submitted to Canonical Discrimination analysis for each task to reduce dimensionality and assess relatedness. The graphs show score plots for CanDisc factor-1 versus factor-2, the scatters are the mice, and the circle is the 90% confidence interval (CI) for prediction of group membership.

A: Free adaptation; **B:** nosepoke adaptation, **C:** Place preference learning (PPL1); **D:** Place preference learning reversal (PPL2r); **E:** place preference learning (PPL3).

Suppl. Figure S9

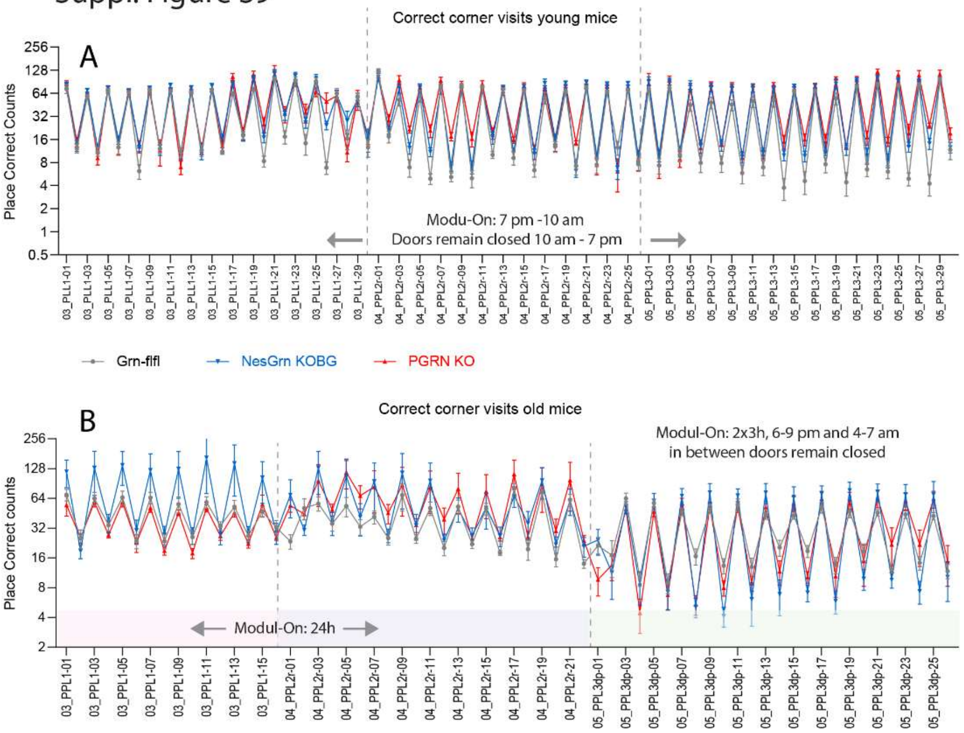

Suppl. Figure S9

Time course of absolute correct corner visits of young and old mice
A: Counts of correct visits per hour (Visits /h) during different tasks in IntelliCages in **young** mice
The data are the means ± sem of n = 8-9 female mice per group.
B: Counts of correct visits per hour (Visits /h) during different tasks in IntelliCages in **old** mice
The data show means ± sem of 12, 10 and 6 female Grn-flfl, NesGrn KOBG and PGRN KO mice

Reference

1. Karperien AL, Jelinek HF: **Morphology and Fractal-Based Classifications of Neurons and Microglia in Two and Three Dimensions.** *Adv Neurobiol* 2024, **36:**149-172.
