# Supplementary material for: Selective neuronal restoration of progranulin does not prevent the frontotemporal dementia like-phenotype of progranulin knockout mice
Source: J Neuroinflammation. 2026 Jan 10;23:34. doi: 10.1186/s12974-025-03665-3 (PMC12836895; doi:10.1186/s12974-025-03665-3)
Supplement: Supplementary file 4 — Supplementary Material 4. [file 12974_2025_3665_MOESM4_ESM.pdf]

Suppl. Histology IBA1 - GFAP

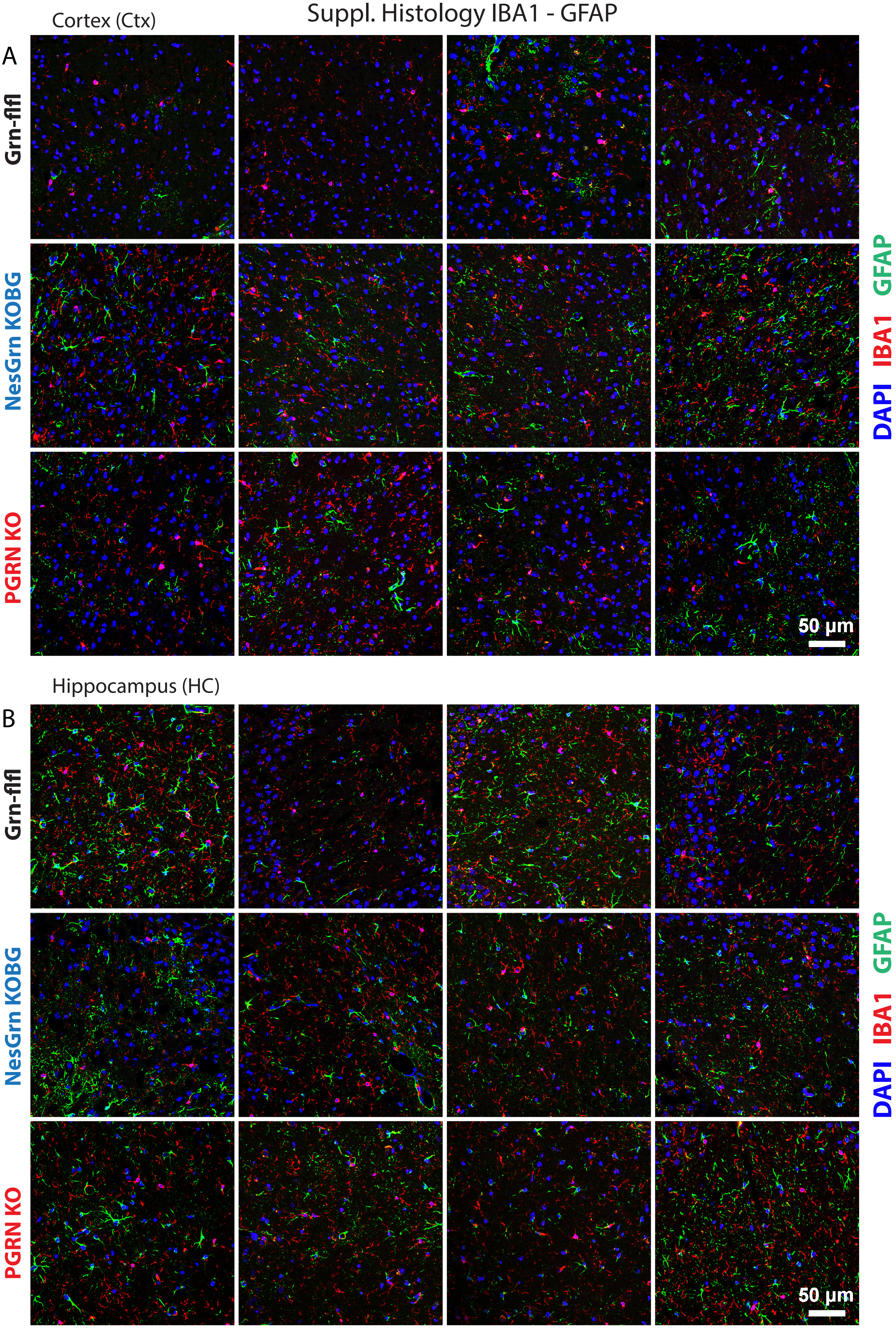

Suppl. Histology IBA1 - GFAP

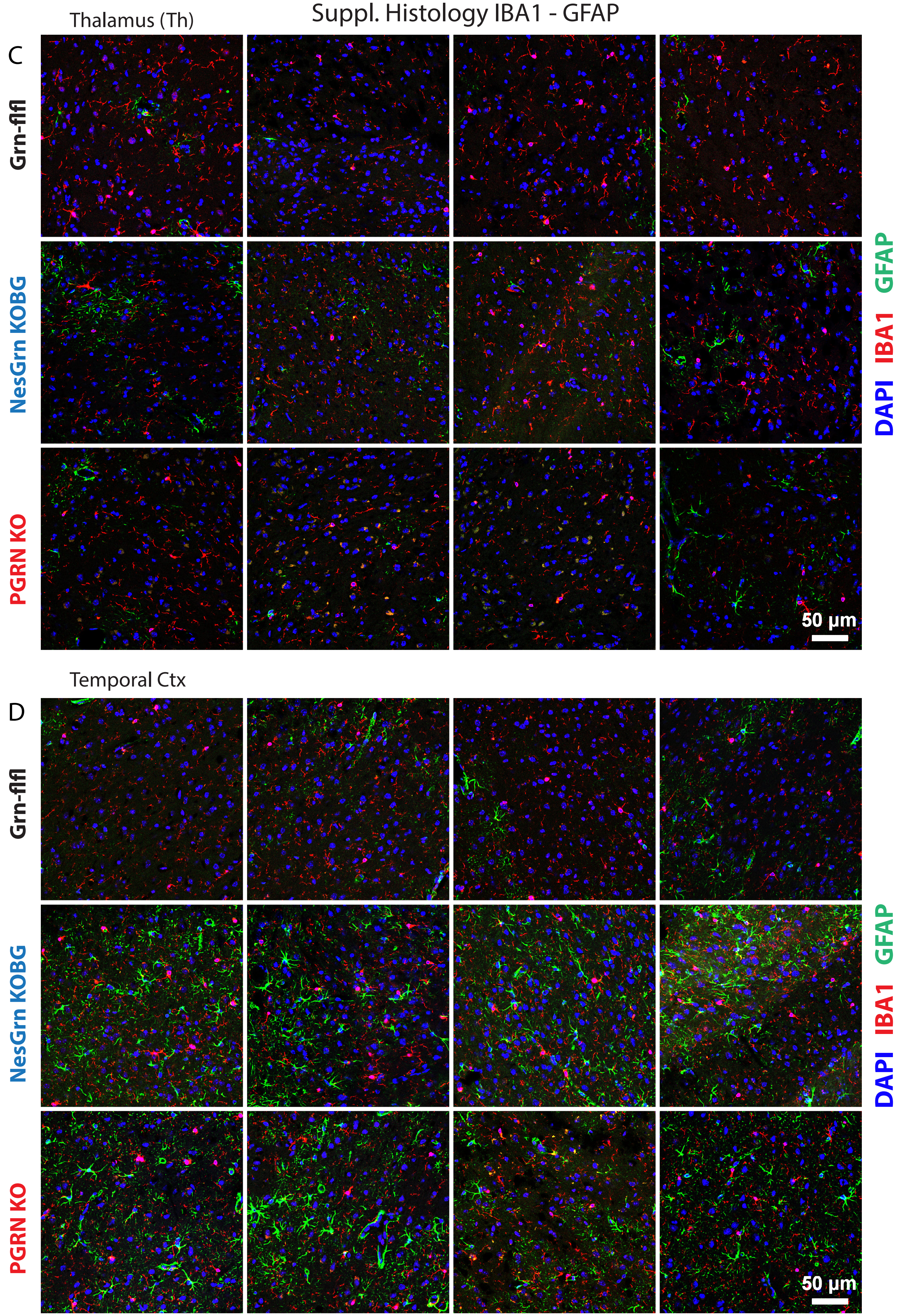

Suppl. Histology CD11b - CD68

Cortex (Ctx)

A

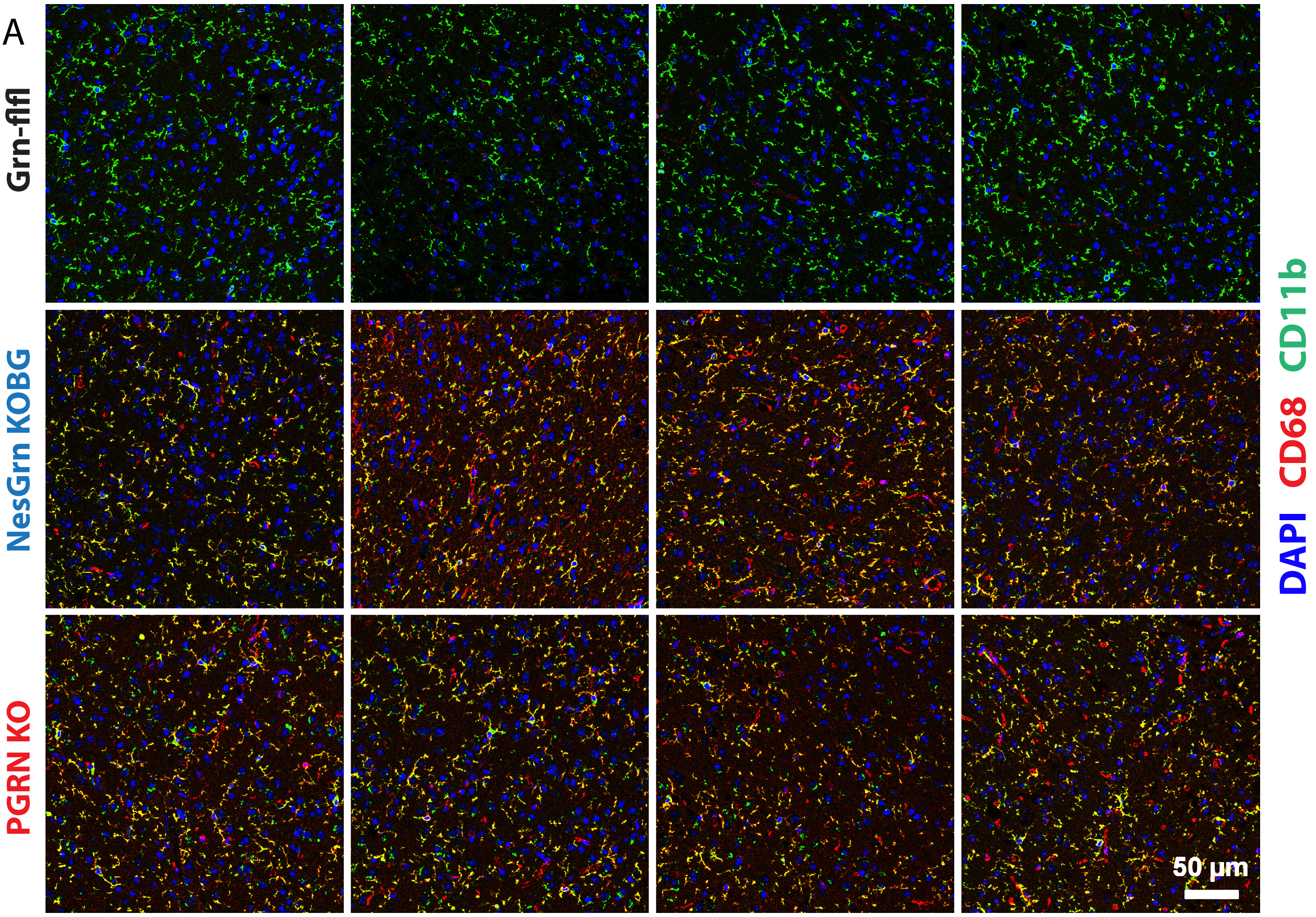

Hippocampus (HC)

B

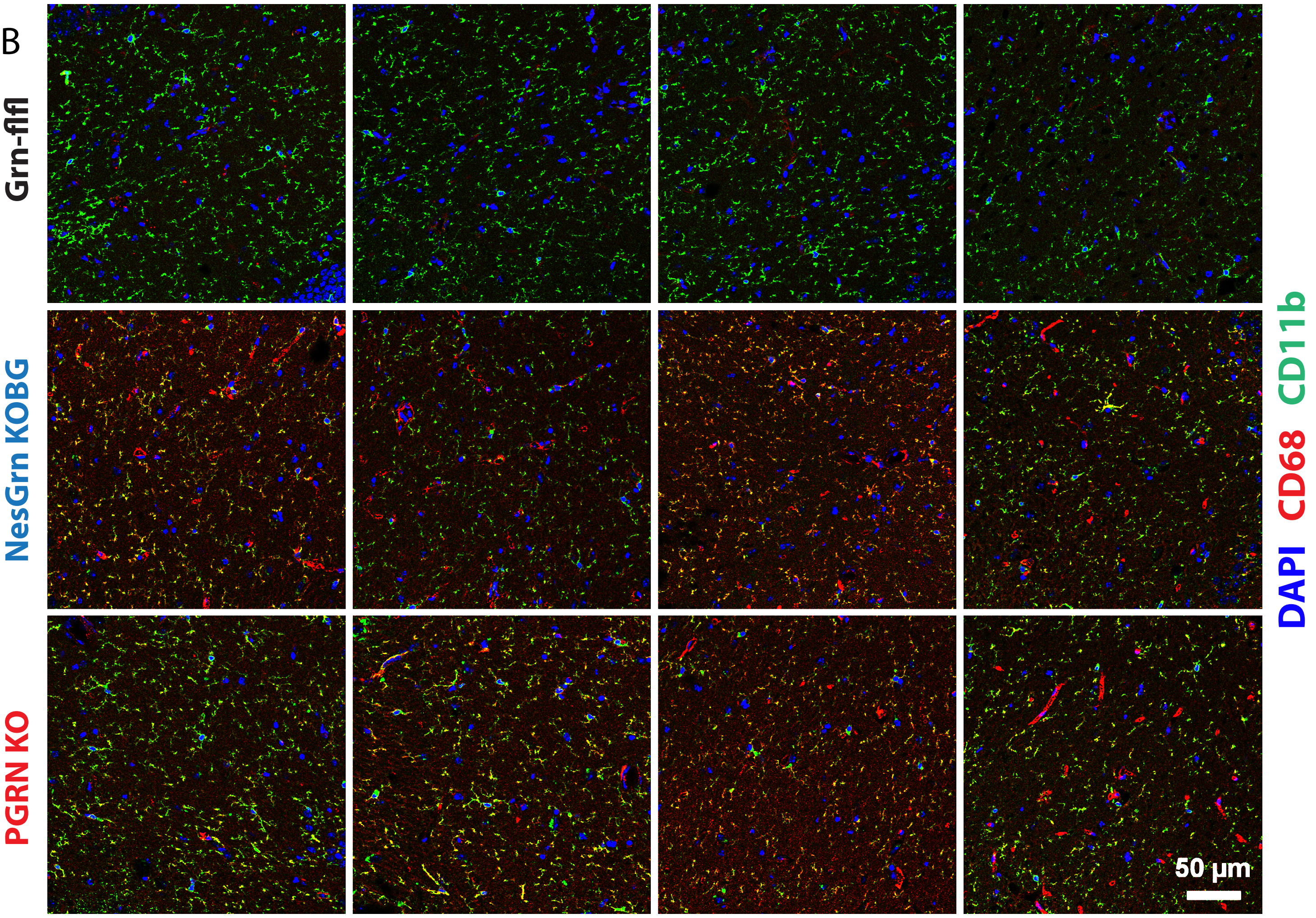

Suppl. Histology CD11b - CD68

Thalamus (Th)

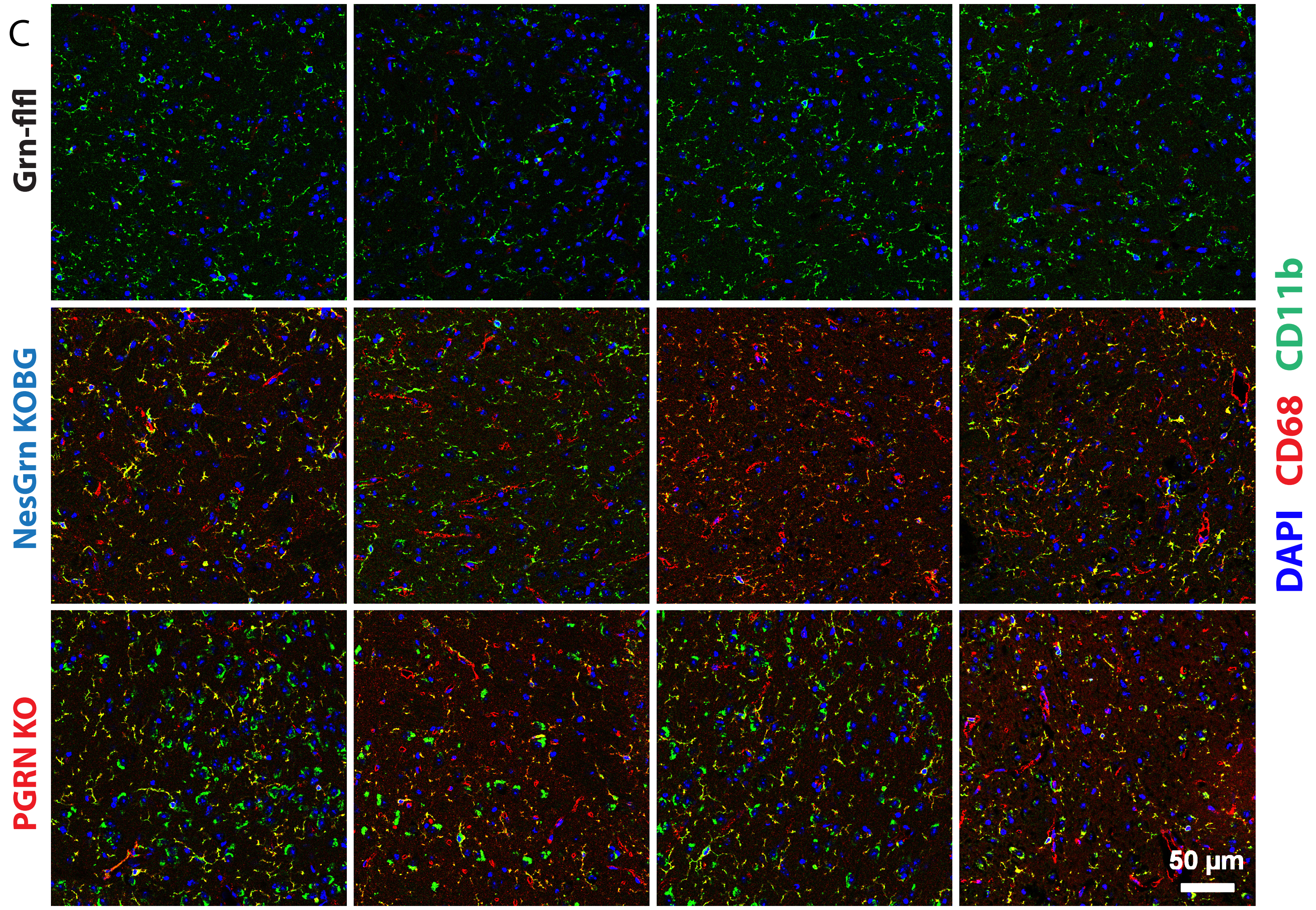

Temporal Ctx

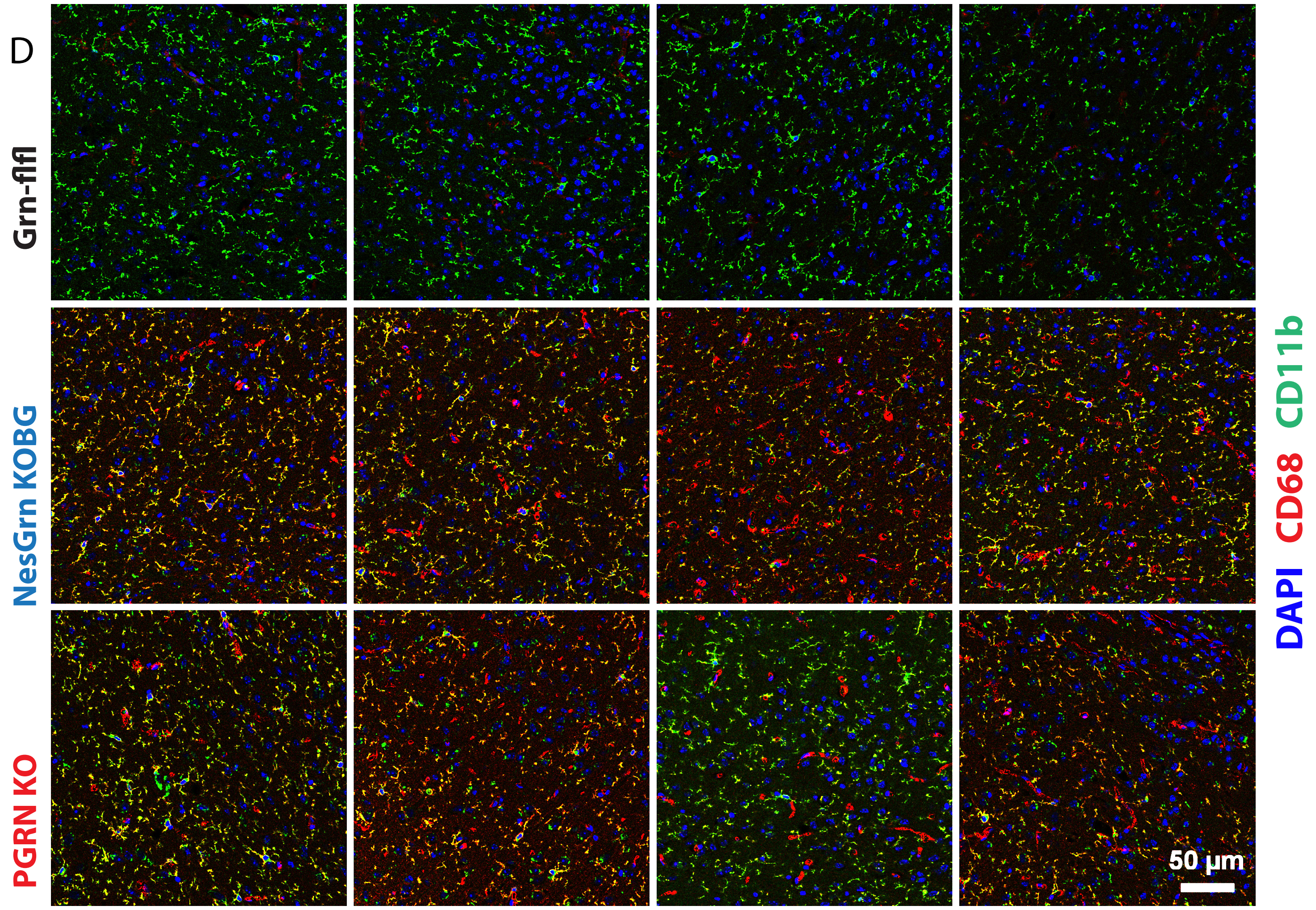

Microgliosis and astrogliosis in four regions of the mouse brain

Suppl. histology IBA1 - GFAP A-D: Immunofluorescent images of the motor cortex (A), hippocampus (B), thalamus (C) and temporal cortex (D) from old Grn-flfl, NesGrn KOBG and PGRN KO mice showing IBA1 immunoreactive microglia, GFAP immunoreactive astrocytes and DAPI as nuclear counterstain. Overviews indicating the sites and quantification are shown in the main body in Figure 3. For each genotype, the images show 4 examples of 3-5 mice per genotype.

Suppl. histology of CD11b - CD68 A-D: In analogy to IBA1/GFAP the images show microglia activity marker CD11 and CD68 expression in the motor cortex (A), hippocampus (B), thalamus (C) and temporal cortex (D).
